# Genome-wide identification of inter-individually variable DNA methylation sites improves the efficacy of epigenetic association studies

**DOI:** 10.1038/s41525-017-0016-5

**Published:** 2017-04-13

**Authors:** Tsuyoshi Hachiya, Ryohei Furukawa, Yuh Shiwa, Hideki Ohmomo, Kanako Ono, Fumiki Katsuoka, Masao Nagasaki, Jun Yasuda, Nobuo Fuse, Kengo Kinoshita, Masayuki Yamamoto, Kozo Tanno, Mamoru Satoh, Ryujin Endo, Makoto Sasaki, Kiyomi Sakata, Seiichiro Kobayashi, Kuniaki Ogasawara, Jiro Hitomi, Kenji Sobue, Atsushi Shimizu

**Affiliations:** 1 Division of Biomedical Information Analysis, Iwate Tohoku Medical Megabank Organisation, Disaster Reconstruction Center, Iwate Medical University, 2-1-1 Nishitokuta, Yahaba, Shiwa, Iwate 028-3694 Japan; 2 Division of Biobank and Data Management, Iwate Tohoku Medical Megabank Organisation, Disaster Reconstruction Center, Iwate Medical University, 2-1-1 Nishitokuta, Yahaba, Shiwa, Iwate 028-3694 Japan; 3 Department of Integrative Genomics, Tohoku Medical Megabank Organisation, Tohoku University, 2-1 Seiryo, Aoba, Sendai 980-8573 Japan; 4 Graduate School of Information Sciences, Tohoku University, 6-3-09, Aramaki-aza Aoba, Aoba-ku, Sendai, Miyagi 980-8597 Japan; 5 Department of Medical Biochemistry, Tohoku University Graduate School of Medicine, 2-1 Seiryo, Aoba, Sendai 980-8575 Japan; 6 Division of Clinical Research and Epidemiology, Iwate Tohoku Medical Megabank Organisation, Disaster Reconstruction Center, Iwate Medical University, 2-1-1 Nishitokuta, Yahaba, Shiwa, Iwate 028-3694 Japan; 7 Department of Hygiene and Preventive Medicine, School of Medicine, Iwate Medical University, 19-1 Uchimaru, Morioka, Iwate 020-8505 Japan; 8 Division of Community Medical Supports and Health Record Informatics, Iwate Tohoku Medical Megabank Organisation, Disaster Reconstruction Center, Iwate Medical University, 2-1-1 Nishitokuta, Yahaba, Shiwa, Iwate 028-3694 Japan; 9 Division of Biomedical Information Analysis, Institute for Biomedical Sciences, Iwate Medical University, 2-1-1 Nishitokuta, Yahaba, Shiwa, Iwate 028-3694 Japan; 10 Division of Public Relations and Planning, Iwate Tohoku Medical Megabank Organisation, Disaster Reconstruction Center, Iwate Medical University, 2-1-1 Nishitokuta, Yahaba, Shiwa, Iwate 028-3694 Japan; 11 Department of Internal Medicine, School of Medicine, Iwate Medical University, 19-1 Uchimaru, Morioka, Iwate 020-8505 Japan; 12 Iwate Tohoku Medical Megabank Organisation, Disaster Reconstruction Center, Iwate Medical University, 2-1-1 Nishitokuta, Yahaba, Shiwa, Iwate 028-3694 Japan; 13 Division of Ultrahigh Field MRI, Institute for Biomedical Sciences, Iwate Medical University, 2-1-1 Nishitokuta, Yahaba, Shiwa, Iwate 028-3694 Japan; 14 Department of Neurosurgery, School of Medicine, Iwate Medical University, 19-1 Uchimaru, Morioka, Iwate 020-8505 Japan; 15 Department of Anatomy, School of Medicine, Iwate Medical University, 2-1-1 Nishitokuta, Yahaba, Shiwa, Iwate 028-3694 Japan

## Abstract

Epigenome-wide association studies, which searches for blood-based DNA methylation signatures associated with environmental exposures and/or disease susceptibilities, is a promising approach to a better understanding of the molecular aetiology of common diseases. To carry out large-scale epigenome-wide association studies while avoiding false negative detection, an efficient strategy to determine target CpG sites for microarray-based or sequencing-based DNA methylation profiling is essentially needed. Here, we propose and validate a hypothesis that a strategy focusing on CpG sites with high DNA methylation level variability may attain an improved efficacy. Through whole-genome bisulfite sequencing of purified blood cells collected from > 100 apparently healthy subjects, we identified ~2.0 million inter-individually variable CpG sites as potential targets. The efficacy of our strategy was estimated to be 3.7-fold higher than that of the most frequently used strategy. Our catalogue of inter-individually variable CpG sites will accelerate the discovery of clinically relevant DNA methylation biomarkers in future epigenome-wide association studies.

## Epigenomics: Finding DNA modifications that predict disease

Patterns of chemical modifications on DNA can predict the risk of certain diseases, but the challenge is knowing where to look for them. Atsushi Shimizu from Iwate Medical University in Japan and his colleagues determined the location of these modifications, methyl groups added to DNA’s cytosine bases, in over hundred healthy people and found two million regions that vary widely between individuals. They used previous data to show that these regions are far more likely to change during the course of disease than genomic loci that are unchanged in all subjects. Current methods to look for disease-specific methylation changes can only profile at most 20% of the genome and Shimizu’s work will help focus on the 20% that matter for identifying disease risk and lead to better diagnosis and prognosis.

Emerging evidence shows that epigenetic signatures in blood cells are influenced by genetic variants, are altered by environmental exposures, and are linked to diseases susceptibilities.^1–4^ Accordingly, searching for epigenetic signatures associated with exposures and diseases is a promising approach to a better understanding of the molecular aetiology of common diseases, which are attributable to both genetic and environmental factors.^5, 6^ From this perspective, locus-specific DNA methylation (DNAm) signatures in blood cells have been intensively associated with various exposures, intermediate phenotypes, and diseases, including tobacco smoking,^7, 8^ arsenic exposure,^9^ blood pressure,^4^ body mass index (BMI),^10–12^ immunoglobulin E,^13^ type 2 diabetes,^14–16^ rheumatoid arthritis,^2^ lung cancer^17^ and schizophrenia,^18^ through epigenome-wide association studies (EWASs). Prospective studies revealed that DNAm signatures of pre-disease subjects are distinguishable from those of healthy subjects and therefore, are useful for identifying persons at high risk.^11, 14, 17^ In addition, we and others have proven DNAm to have desirable biomarker features, i.e., high measurement accuracy,^19–21^ high chemical stability during sample transportation,^22^ and high biological stability against frequent immunological stimuli.^23^ Hence, locus-specific DNAm signatures are becoming a new fascinating tool for biomarker discovery.^1, 3, 24^

Currently, in the discovery step of almost all EWASs, the Illumina HumanMethylation27^25^ or HumanMethylation450 (HM450)^19, 26^ microarray is used to profile DNAm levels of 27 thousand or 480 thousand CpG sites, respectively.^7–18^ As the human reference genome (hg19) harbours 26.8 million autosomal CpGs, only ~2% or less of human autosomal CpG sites are probed by these microarrays. Recently, the MethylationEPIC microarray^20^ (Illumina) has become available, which allows measuring the DNAm levels of 850 thousand CpGs (~3%). For higher CpG coverage, sequencing-based profiling methods are available. In reduced representation bisulfite sequencing (RRBS),^27^ genomic DNA is digested with the methylation-insensitive restriction enzyme *Msp*I followed by fragment size selection, adaptor ligation, bisulfite treatment, and massively parallel sequencing. Typically, ~10% of the CpGs in the human genome are interrogated by RRBS.^28^ Methyl-capture sequencing systems, such as SureSelect Human Methyl-Seq^29^ (Agilent Technologies) and SeqCap Epi CpGiant^30^ (Roche NimbleGen), use oligonucleotide probes designed to hybridise target regions of interest. The SureSelect panel covers ~3.7 million CpG sites (~13%)^28^ while CpGiant measures ~5.5 million CpGs (~20%).^28^ With ~90% coverage of human CpGs, whole-genome bisulfite sequencing (WGBS) provides the highest coverage among the currently available DNAm profiling technologies.^31–33^ However, because of its high cost, it is presently infeasible to apply WGBS to large-scale EWASs, which require DNAm profiling of hundreds or thousands of subjects.^7, 8, 10–12, 14–16, 34^ Therefore, microarrays and targeted bisulfite sequencing are currently practicable for large-scale EWASs and thus, effective strategies to select target regions are essentially needed to improve the efficacy of epigenetic association studies.

Microarray and methyl-capture sequencing probes have been designed for multiple purposes, including studies on cancer tissues,^35^ studies on the cell-type specificity of epigenetic signatures,^36^ and blood-based EWASs.^7–18^ All probe designs targeted CpG island (CGI) and promoter regions^19, 26^ as these regions are involved in epigenetic regulation of gene expression.^37, 38^ RRBS is also likely to measure CpGs in CGIs and promoters because the *Msp*I cleavage site (CCGG) is over-represented in those regions.^27^ As previous studies have shown that DNAm levels in CGI shores vary among tumour tissue types,^39^ CGI shores have been included as probe-set targets. Furthermore, functional DNA elements, such as DNase I-hypersensitive sites (DHSs), transcription factor binding sites (TFBSs), and active histone modifications, have been genome widely mapped.^37, 40^ Accordingly, CpG sites located at those functional DNA elements have been included in probe sets for DNAm profiling.^19, 26^ It is noteworthy that evidence for target CpG sites was derived mainly from studies on the cell-type specificity of epigenetic signatures and studies on cancer tissues rather than from studies on inter-individual differences in epigenetic signatures of blood cells. Thus, the multipurpose designs of the probe sets for DNAm profiling may not be optimal for blood-based EWASs. Indeed, previous epigenetic association studies have revealed that DNAm levels measured with microarrays are invariable for most CpG sites in the study populations.^41, 42^ As invariable DNAm signatures cannot be associated with exposures, intermediate phenotypes, or diseases, current designs of probe sets are inefficient for blood-based EWASs.

We considered that a strategy focusing on inter-individually variable CpG sites may improve the efficacy of epigenetic association studies. Hence, we hypothesised that common DNAm variations (CDMV) are more likely to be associated with environmental exposures or biomedical traits than rare DNAm variations. To test this hypothesis, which we referred to as ‘CDMV hypothesis’, we genome widely identified inter-individually variable CpG sites and evaluated the efficacy of a strategy to select target CpG sites based on the CDMV hypothesis (referred to as ‘CDMV strategy’). Through large-scale sequencing, comprehensive DNAm profiling, and statistical data analyses, we showed the validity of the CDMV hypothesis and provided proof-of-concept of the improved efficacy of the CDMV strategy.

## Results

### Study design

We aimed to genome widely identify inter-individually variable CpG sites, validate the CDMV hypothesis, and evaluate the efficacy of the CDMV strategy. To these ends, we designed our study in terms of study population, target blood cells, DNAm profiling method and statistical analyses, as follows.

#### Study population

To minimise potential selection bias, we used a population-based design, enrolling apparently healthy adults from residents of the Iwate prefecture, Japan.

#### Target blood cells

**Fig. 1:**
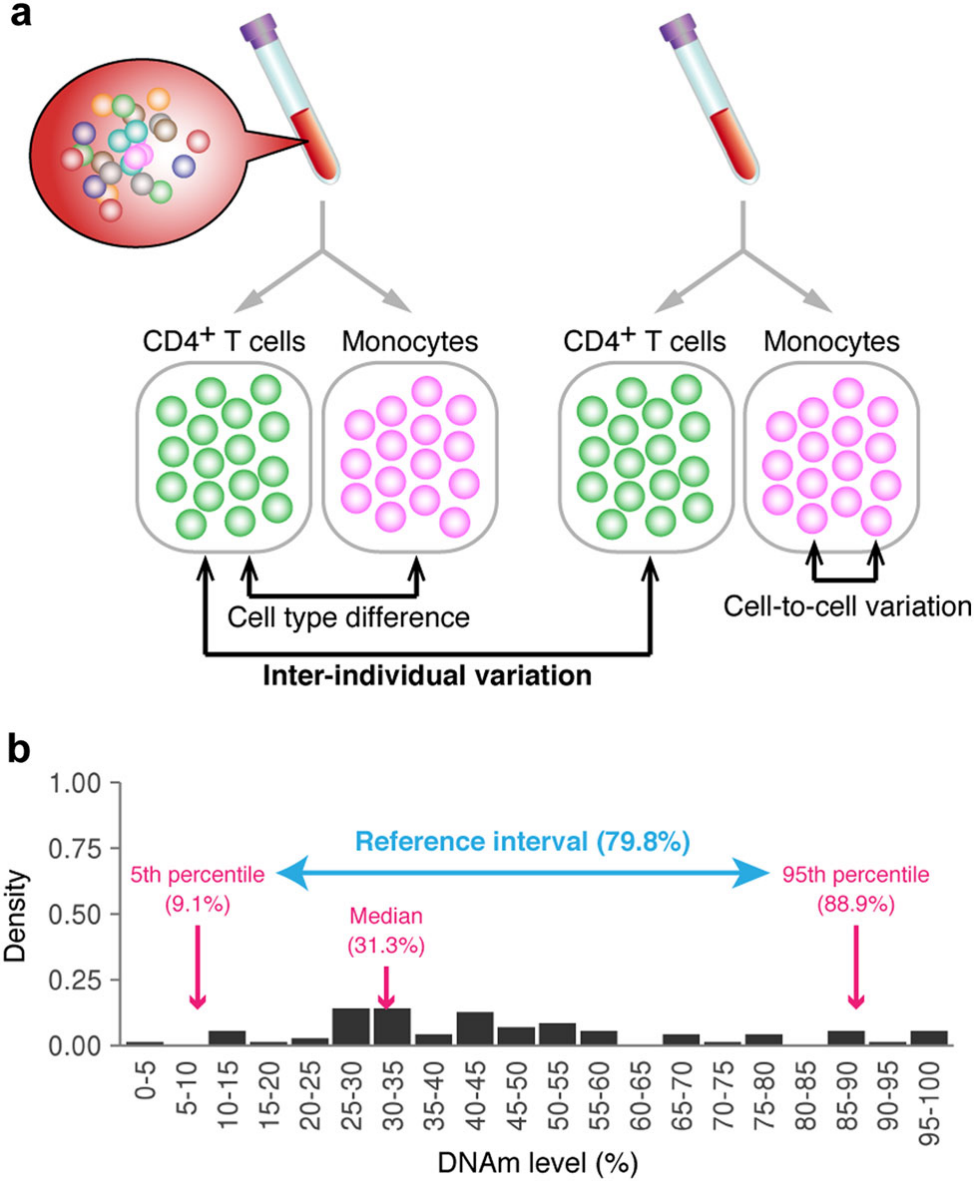
DNAm variations in purified blood cells. **a** Schematic representation of sources of DNAm variation in blood cells. **b** Definition of reference interval as an indicator of inter-individual DNAm variability. The reference interval for a CpG site was defined as the difference between the 95th and 5th percentiles of DNAm levels across individuals

DNAm variations include differences between distinct cell types, inter-individual variations within a cell type, and cell-to-cell variations within a cell type and individual (Fig. 1a). Because we aimed to identify inter-individually variable CpG sites, we focused on inter-individual DNAm variations within a cell type. Therefore, we analysed purified blood cells rather than whole blood or peripheral blood mononuclear cells (PBMCs). Concretely, we selected classical CD14^++^/CD16^−^ monocytes and CD4^+^ T cells. Human monocytes consist of three subsets, which can be distinguished by surface expression of CD14 and CD16.^43^ Classical monocytes, the major subset constituting 5–10% of leucocytes,^43^ are a homogeneous and therefore desirable population for analysing inter-individual DNAm variation within a cell type. In addition, monocytes play a key role in the innate immune system including pathogen surveillance, phagocytosis, and antigen presentation.^44^ Monocyte-specific DNAm signatures have been associated with type 1 diabetes^45^ and smoking exposure.^46^ CD4^+^ T cells make up a large fraction of lymphocytes (27–58%)^47^ and play a central role in the adaptive immune system, namely in antigen recognition, activation of other immune cells, and immune response regulation.^48^ Contrary to classical monocytes, they are composed of several subsets, including naive CD4^+^ T (Th0), T helper 1, T helper 2 (Th2) and regulatory T cells.^49^ Accordingly, inter-individual DNAm variation observed in CD4^+^ T cells includes subset-specific DNAm variation. Regardless of the heterogeneity, we included CD4^+^ T cells in our study because they were used in a number of EWASs that reported locus-specific DNAm signatures in these cells associated with BMI,^12^ waist circumstance,^12^ and blood lipid level.^50^

#### DNAm profiling method

To genome-widely identify inter-individually variable CpG sites, we carried out WGBS for comprehensive DNAm profiling. In addition to WGBS, we used whole-genome sequencing (WGS) and RNA sequencing (RNA-Seq) for the profiling of genomic variants and gene expressions, respectively.

#### Statistical analyses

We estimated the DNAm variability for each CpG site by means of the *reference interval*, which is here defined as the difference between the 95th and 5th percentiles of the DNAm levels across individuals (Fig. 1b). To test the CDMV hypothesis and evaluate the efficacy of the CDMV strategy, we defined the *biomarker likelihood* for a group of CpG sites as the number of CpGs in the group that were associated with any environmental exposures and/or biomedical traits in previous EWASs divided by the number of total CpGs in the group. To test the CDMV hypothesis, we compared the biomarker likelihood between narrow and broad reference interval groups. To evaluate the efficacy of the CDMV strategy, we selected CpG sites exhibiting broad reference intervals and estimated the degree of improvement in efficacy by comparing the biomarker likelihood for CpGs selected by the CDMV strategy with that for CpGs targeted by existing probe sets.

### Comprehensive DNAm profiling by WGBS

In total, 109 apparently healthy subjects between the ages of 34 and 74 years from residents of the Iwate prefecture, Japan, were enrolled (Table 1; Supplementary Table 1). Classical CD14^++^/CD16^−^ monocytes and CD4^+^ T cells were isolated by fluorescence-activated cell sorting (FACS) with high purities (Supplementary Table 2). We subjected 102 samples to WGBS-based profiling of monocytes and CD4^+^ T cells. Both cell types were obtained from the same individual in 95 instances. The mean age of the subjects donating monocytes was 58.5 years and that of CD4^+^ T donors was 58.0 years. The number of males among monocyte and CD4^+^ T donors was 48 (47.1%) and 49 (48.0%), respectively.

**Table 1:** Statistics for WGBS-based DNAm profiles

		Monocytes (*N* = 102)	CD4^+^ T cells (*N* = 102)
Subjects	Males, *N* (%)	48 (47.1)	49 (48.0)
	Age, year^a^	58.5 ± 11.0	58.0 ± 11.4
Sequencing statistics	Number of raw reads^a^	780,709,034 ± 45,934,514	779,212,752 ± 40,833,955
	Number of raw bases^a^	97,588,629,235 ± 5,741,814,291	97,401,593,968 ± 5,104,244,342
	Raw depth^a^	31.1 ± 1.8	31.0 ± 1.6
	Number of reads after quality-control filtering^a^	624,432,868 ± 38,766,158	667,934,331 ± 33,002,407
	Number of bases after quality-control filtering^a^	47,245,324,384 ± 3,327,997,912	52,472,000,722 ± 2,376,329,508
	Depth after quality-control filtering^a^	15.1 ± 1.1	16.7 ± 0.8
CpG statistics	Number of autosomal CpGs in the human genome	26,752,702	26,752,702
	Number of autosomal CpGs covered by at least 1 read	24,932,694 ± 163,384	24,939,224 ± 121,488
	Percentage of autosomal CpGs covered by at least 1 read	93.2 ± 0.6	93.2 ± 0.5
	Number of autosomal CpGs after depth filtering	23,404,723 ± 362,243	23,584,230 ± 238,187
	Percentage of autosomal CpGs after depth filtering	87.5 ± 1.4	88.2 ± 0.9
	Number of autosomal CpGs with a call rate of ≥ 50%	23,941,843	24,037,541
	Percentage of autosomal CpGs with a call rate of ≥ 50%	89.5	89.9

^a^ Average ± standard deviation

*WGBS* whole-genome bisulfite sequencing

In total, 159.1 billion reads and 19.9 tera base pairs of sequences were generated by WGBS (Table 1; Supplementary Tables 3 and 4). The average raw read depth was 31.1 for monocytes and 31.0 for CD4^+^ T cells, satisfying read depth recommendations for WGBS analysis.^51^ Bioinformatics processing and quality-control filtering resulted in DNAm profiles consisting of 23.9 million autosomal CpGs for monocytes and 24.0 million autosomal CpGs for CD4^+^ T cells. We only included CpGs that occurred in the human reference sequence. To minimise the effects of genetic variants on reference interval estimates, for each CpG site in the reference, when genetic variants altered the CpG sequence for part of the subjects, the DNAm level for the subjects was considered a missing value. The DNAm profiles comprehensively covered ~90% of autosomal CpGs in the human genome. Summary statistics for WGS and RNA-Seq data are presented in Supplementary Tables 5 and 6. WGS data were available for 105 out of the 109 participants.

**Fig. 2:**
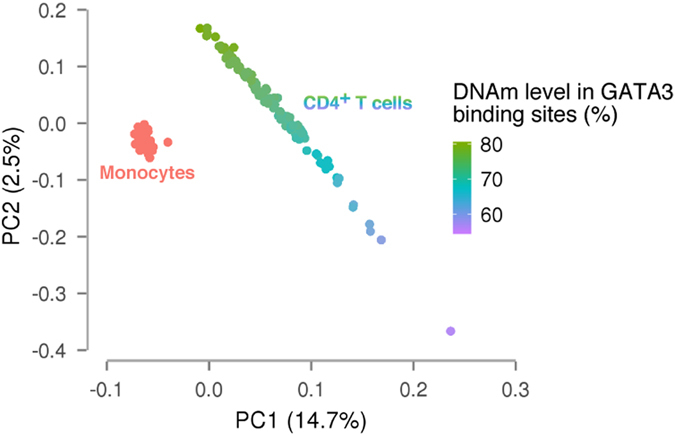
PC analysis of comprehensive DNAm profiles. The *x*-axis and *y*-axis represent the first and second PC, respectively. Monocytes are indicated in *red*, while CD4^+^ T cells are indicated with a colour gradient according to the median DNAm level of GATA3 binding sites, ranging from low (*purple*) to high (*green*). GATA3 is involved in the specification of naive CD4^+^ T cells to Th2 cells and therefore, the median DNAm level of GATA3 binding sites is expectedly negatively correlated with the proportion of Th2 cells

Based on the DNAm profiles of ~24 million CpGs, the average DNAm level was 80.4% for monocytes and 79.0% for CD4^+^ T cells. Principal component (PC) analysis using the DNAm profiles of the ~24 million CpGs showed that monocytes and CD4^+^ T cells were evidently segregated by PC1 (Fig. 2). Compared to CD4^+^ T cells, monocytes were densely clustered, both in PC1 and PC2. The wider distribution of CD4^+^ T cells was attributable to the variation in the composition of T cell subsets (Fig. 2). These results suggested that DNAm variation between the two cell types and that attributable to T cell subsets was larger than inter-individual DNAm variation within a cell type, consistent with a previous study.^52^ This finding highlighted the importance of using purified blood cells for distinguishing inter-individual DNAm variation from cell type-specific DNAm differences.

### Validity of the CDMV hypothesis: inter-individually variable CpG sites tended to have been associated in previous EWASs

**Fig. 3:**
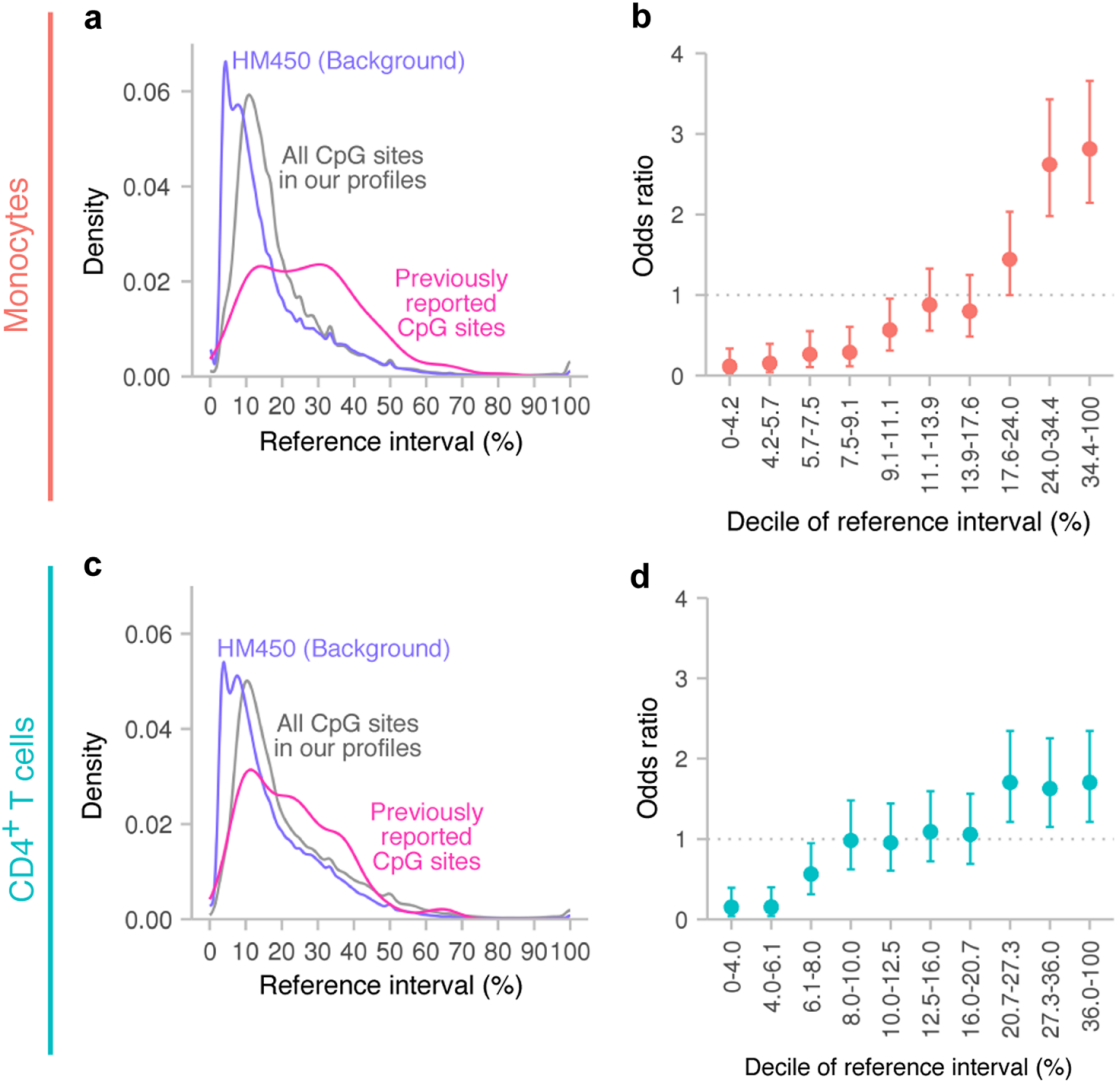
Validity of the CDMV hypothesis. **a** Reference interval distributions in monocytes. The reference interval distribution for the ~24 million CpG sites included in our comprehensive profiles is indicated in *grey*. The reference interval distribution for CpG sites probed by HumanMethylation450 (HM450) is shown in *purple*. The reference interval distribution for CpG sites previously reported by blood-based EWASs is represented in *magenta*. **b** OR in monocytes. CpG sites probed by HM450 are categorized into ten groups according to reference interval deciles. The OR was estimated by comparing biomarker likelihood for each group of CpG sites with that for all CpG sites probed by HM450 (average biomarker likelihood). Labels in the *x*-axis represent ranges of DNAm levels as a percentage for each decile. The 95% CIs are shown as *solid lines*. **c** Reference interval distributions in CD4^+^ T cells. **d** OR in CD4^+^ T cells

We estimated reference intervals for each of the ~24 million CpG sites that passed our quality filter. The distributions of the reference intervals were unimodal, peaking at ~11% for both monocytes and CD4^+^ T cells (Fig. 3a, c).

To validate the CDMV hypothesis, we systematically surveyed published EWASs that used the HM450 microarray in the discovery step and validated candidate CpG sites in independent samples. In total, 269 CpG sites were reported from 11 EWASs on tobacco smoking,^7, 8^ obesity,^10–12^ type 2 diabetes,^14–16^ lipid levels,^50, 53^ and schizophrenia^18^ (Supplementary Tables 7 and 8). Almost all (99.3%) previously reported CpG sites had been identified from whole blood samples and others had been derived from purified CD4^+^ T cells. A majority (83.6%) of CpG sites had been associated in case-control studies and others had been identified in population-based studies. Almost all (99.3%) CpG sites had been discovered in EWASs of Caucasians or African Americans and others had been derived from EWASs of Indian Asians.

In monocytes, compared to the background reference interval distributions for the CpG sites probed by HM450, the CpG sites associated in previous EWASs clearly exhibited larger reference intervals (Fig. 3a). The median reference interval for the background CpG sites was 11.1%, whereas the median reference interval for the associated CpG sites was 26.5%. The biomarker likelihood increased with broadening of the reference interval of the CpG site (Fig. 3b). Compared to the average likelihood, the CpGs in the narrowest reference interval decile had a 9.1-fold lower likelihood. The CpGs in the broadest reference interval decile had a 2.8-fold higher likelihood. The odds ratio (OR) exceeded 2.0 at the 2 broadest deciles.

In CD4^+^ T cells too, the CpG sites having broad reference intervals tended to have been associated in previous EWASs (Fig. 3c). The OR for the narrowest decile was 0.15 and that for the broadest decile was 1.7. Compared to monocytes, the OR for the broadest decile in CD4^+^ T cells was relatively small. As the distribution of the reference intervals for associated CpG sites was narrower in CD4^+^ T cells (median, 21.0%) than in monocytes (median, 26.5%) (Fig. 3c, d), the difference in OR between the two cell types may arise from cell-specificity of epigenetic signals associated with environmental exposures or biomedical traits. In addition, the background distribution of reference intervals in CD4^+^ T cells (median, 12.5%) was slightly broader than that in monocytes (median, 11.1%). Variations in T cell-subset composition may have inflated the background reference interval levels. Thus, the difference in cell homogeneity between the two cell types may contribute to the difference in OR for the broadest decile.

These results clearly demonstrated the validity of the CDMV hypothesis. Invariable CpG sites were unlikely to have been associated in previous EWASs, whereas inter-individually variable CpG sites tended to have been previously associated.

### Regional analyses of DNAm variability surrounding established DNAm biomarkers

To observe reference intervals surrounding established DNAm biomarkers, we focused on 2 loci harbouring well-established DNAm biomarkers for tobacco smoking: cg05575921^7, 8^ within the aryl-hydrocarbon receptor repressor (*AHRR*) gene and cg03636183^7, 8^ within the thrombin receptor-like 3 (*F2RL3*) gene. These two biomarkers are evidently demethylated in current smokers when compared with never smokers.^7, 8^ A prospective study reported that they were associated with lung cancer risk even after adjustment for smoking status,^17^ implying that epigenetic regulation at these sites may mediate the causal relationship between tobacco smoking and lung cancer. In our DNAm profiles, the associations between the DNAm biomarkers and smoking status were cell type-specific. The associations were significant in monocytes but not in CD4^+^ T cells (Supplementary Table 9). Accordingly, we focused on monocytes for subsequent analyses.

**Fig. 4:**
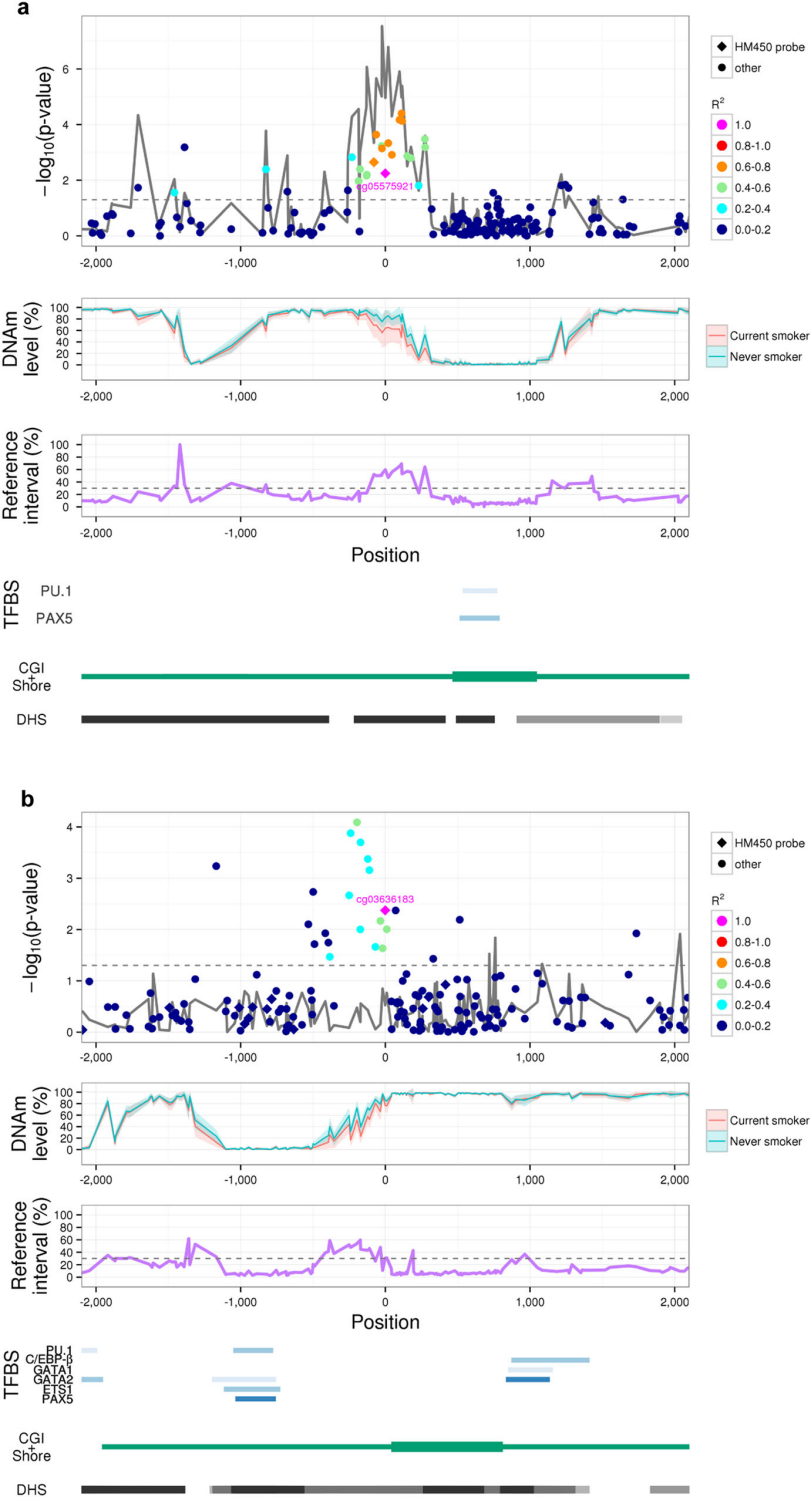
Regional analyses of DNAm variability surrounding established DNAm biomarkers. The *x*-axis indicates the position relative to the DNAm biomarkers cg05575921 in the *AHRR* locus **a** and cg03636183 in the *F2RL3* locus **b**. The *y*-axis represents –log_10_(*P*), where *P* is the *P*-value for associations between DNAm levels and smoking status (*i.e*., current smokers vs. never smokers) (*First panel*). CpGs included in the HM450 probe set are represented by *diamonds*, and other CpGs are represented by *circles*. *Colours* indicate the correlation (*R* ^2^) of the DNAm levels with the biomarker CpGs. The *solid grey line* represents *P-*values for associations between DNAm and gene-expression levels. The *dotted grey line* indicates *P* = 0.05. The *y*-axis reflects the DNAm level(*Second panel*). The *solid red* and *blue lines* indicate the average DNAm levels observed in current and never smokers, respectively. Standard deviations of the DNAm levels are shown as *shaded areas*. The *y*-axis reflects reference intervals (*Third panel*). The *dotted grey line* indicates *y* = 30%. Genomic locations of binding sites for lineage-determining TFs, CGIs, CGI shores and DHSs are shown (*Fourth panel*). *CGI* CpG island, *DNAm* DNA methylation, *DHS* DNase I-hypersensitive site, *HM450* HumanMehylation450, *TFBS* transcription factor binding site

In the *AHRR* locus, the cg05575921 biomarker was located in a CGI shore (Fig. 4a). Two lineage-commitment transcription factors (TFs), PU.1 and PAX5, were found to bind to the CGI. In the binding site, all CpG sites were nearly perfectly demethylated in both current and never smokers, and the reference intervals for the CpG sites were narrow (<20%). The cg05575921 biomarker was located at an intermediately methylated region flanking the TFBS. The biomarker and its surrounding CpG sites exhibited broad reference intervals (>30%) and were associated with both the *AHRR* expression level and smoking status (Fig. 4a; Supplementary Table 9). Genetic variants in this locus were neither associated with the cg05575921 DNAm level nor with smoking status (Supplementary Table 10).

In the *F2RL3* locus, the cg03636183 biomarker was also located in a CGI shore and was flanked by PU.1 and PAX5 binding sites (Fig. 4b). The cg03636183 biomarker and its surrounding CpG sites were intermediately methylated and exhibited broad reference intervals (>30%). DNAm levels at cg03636183 and its surrounding CpG sites were associated with smoking status (Fig. 4b; Supplementary Table 9). Consistent with a previous study,^17^ they were not associated with the *F2RL3* expression level, as this gene is not expressed in blood cells (Supplementary Table 9). Genetic variants in this locus were not associated with the cg03636183 DNAm level or smoking status (Supplementary Table 11).

The fact that the reference intervals surrounding two established DNAm biomarkers were broad (>30%) confirmed the CDMV hypothesis. In addition, these findings suggested that intermediately methylated regions tend to exhibit broad reference intervals and that the presence of regulatory features, such as CGI shores and TFBS flanking regions, relates to the broadness of reference intervals.

### DNAm levels of intermediately methylated CpG sites show inter-individual variability

To test whether intermediately methylated regions indeed associate with broad reference intervals, we stratified the CpG sites by their median DNAm level. Median DNAm levels of ≤20% and ≥80% were categorized as hypomethyalation and hypermethylation, respectively, while levels of 20–80% were considered intermediate methylation. Then, the relationship between DNAm status and reference intervals was investigated based on our comprehensive DNAm profiles covering ~24 million CpG sites.

**Fig. 5:**
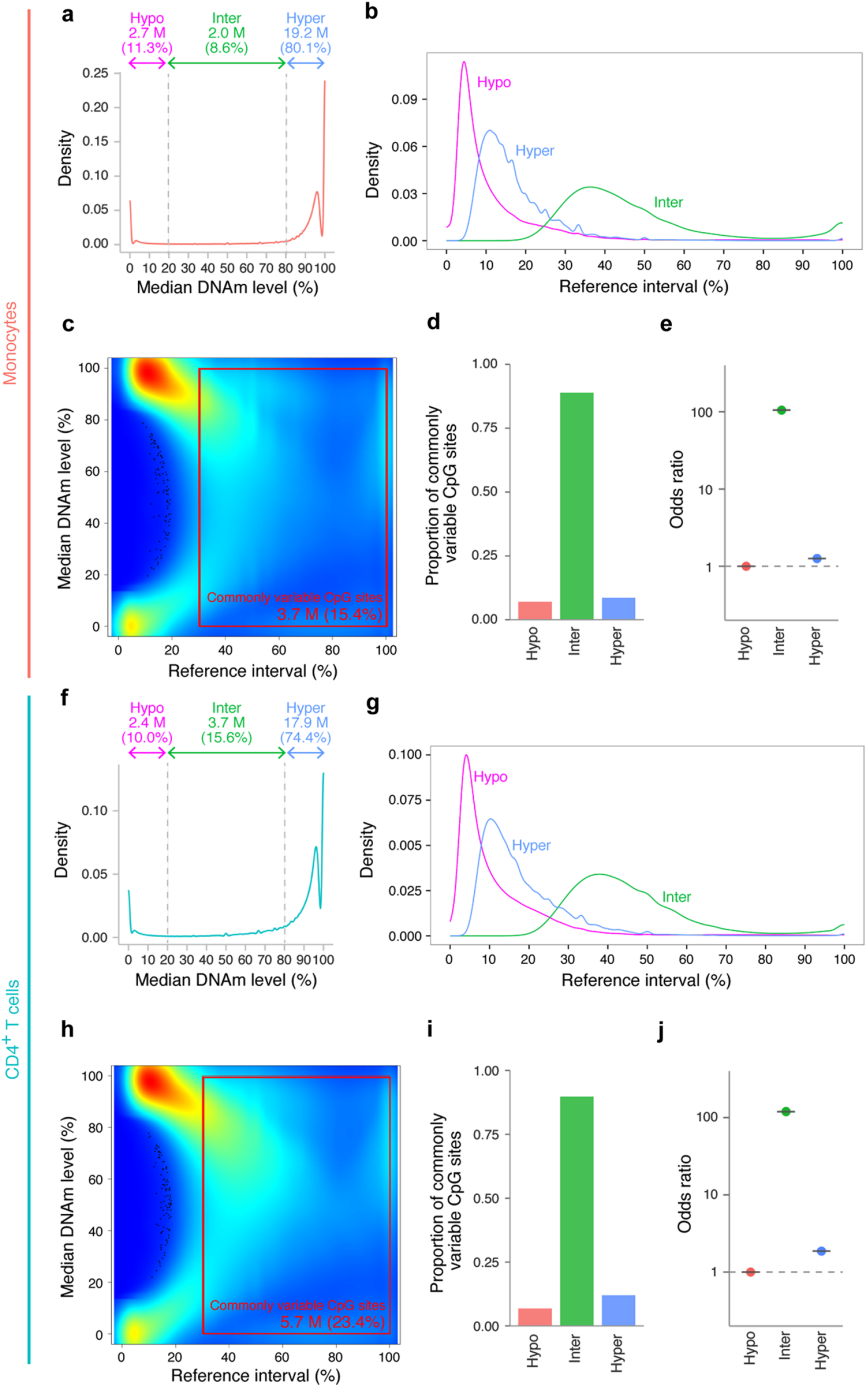
Intermediately methylated CpG sites exhibit large DNAm variability. **a** Distribution of median DNAm levels in monocytes. **b** Distributions of reference intervals in monocytes for hypomethylated (*red*), hypermethylated (*blue*), and intermediately methylated (*green*) CpG sites. **c** CpG-density plot in monocytes. The *x*-axis represents reference intervals, and the *y*-axis indicates median DNAm levels. Density is indicated with a colour gradient, ranging from low (*blue*) to high (*red*). **d** Proportions of commonly variable CpG sites in monocytes. **e** OR in monocytes. The OR was estimated by comparing the proportion of commonly variable CpG sites in hypomethylated, hypermethylated and intermediately methylated CpG sites. The proportion of commonly variable CpG sites in hypomethylated CpG sites is used as the reference. The 95% reference intervals are represented as *black lines*. **f** Density plot of median DNAm level in CD4^+^ T cells. **g** Distribution of reference intervals in CD4^+^ T cells. **h** CpG-density plot in CD4^+^ T cells. **i** Proportions of commonly variable CpG sites in CD4^+^ T cells. **j** OR in CD4+T cells. *Hyper* hypermethylated CpG sites, *Hypo* hypomethylated, *CpG sites* Inter intermediately methylated CpG sites, *M* million

In monocytes, 80.1% of CpG sites were hypermethylated, 11.3% were hypomethylated, and 8.6% were intermediately methylated (Fig. 5a). Large proportions of hypermethylated and hypomethylated CpG sites exhibited narrow reference intervals. The median reference interval for hypomethylated CpGs was 7.1% and that for hypermethylated CpGs was 14.3% (Fig. 5b). Conversely, intermediately methylated CpG sites showed broader reference intervals, with a median of 42.0%. By defining commonly variable CpG sites as those with reference intervals ≥30%, 15.4% of CpG sites were commonly variable in monocytes (Fig. 5c). A majority (88.8%) of intermediately methylated CpG sites were commonly variable, whereas only 7.0% of hypomethylated and 8.7% of hypermethylated CpG sites were commonly variable (Fig. 5d). Compared to hypomethylated sites, intermediately methylated CpG sites showed a 105.0-fold larger fraction of commonly variable CpG sites (Fig. 5e).

In CD4^+^ T cells, similar results were obtained (Fig. 5f–j). Reference intervals for intermediately methylated CpG sites (median, 42.7%) were much broader than those for hypomethylated (7.7%) and hypermethylated (15.0%) CpG sites. Intermediately methylated CpG sites had 119.9 times larger probability to be commonly variable than hypomethylated CpG sites.

These results strongly suggested that intermediately methylated CpG sites exhibit broad reference intervals. No remarkable differences in this regard were observed between the two cell types.

### DNAm signatures at regulatory elements do not show inter-individual variability

We investigated the relationship between genomic regulatory annotations and the broadness of reference intervals. Genomic annotations for promoters, exons and introns were retrieved from the Human GENCODE Gene Set (release 19).^54^ Annotations for CGI and repetitive regions were obtained from the UCSC genome browser.^55^ Genomic intervals for binding sites of 161 TFs, DHSs and 3 types of histone marks—histone H3 acetyl Lys27 (H3K27ac), H3 trimethyl Lys4 (H3K4me3) and H3 monomethyl Lys4 (H3K4me1)—were downloaded from the UCSC ENCODE website.^37, 55^

**Fig. 6:**
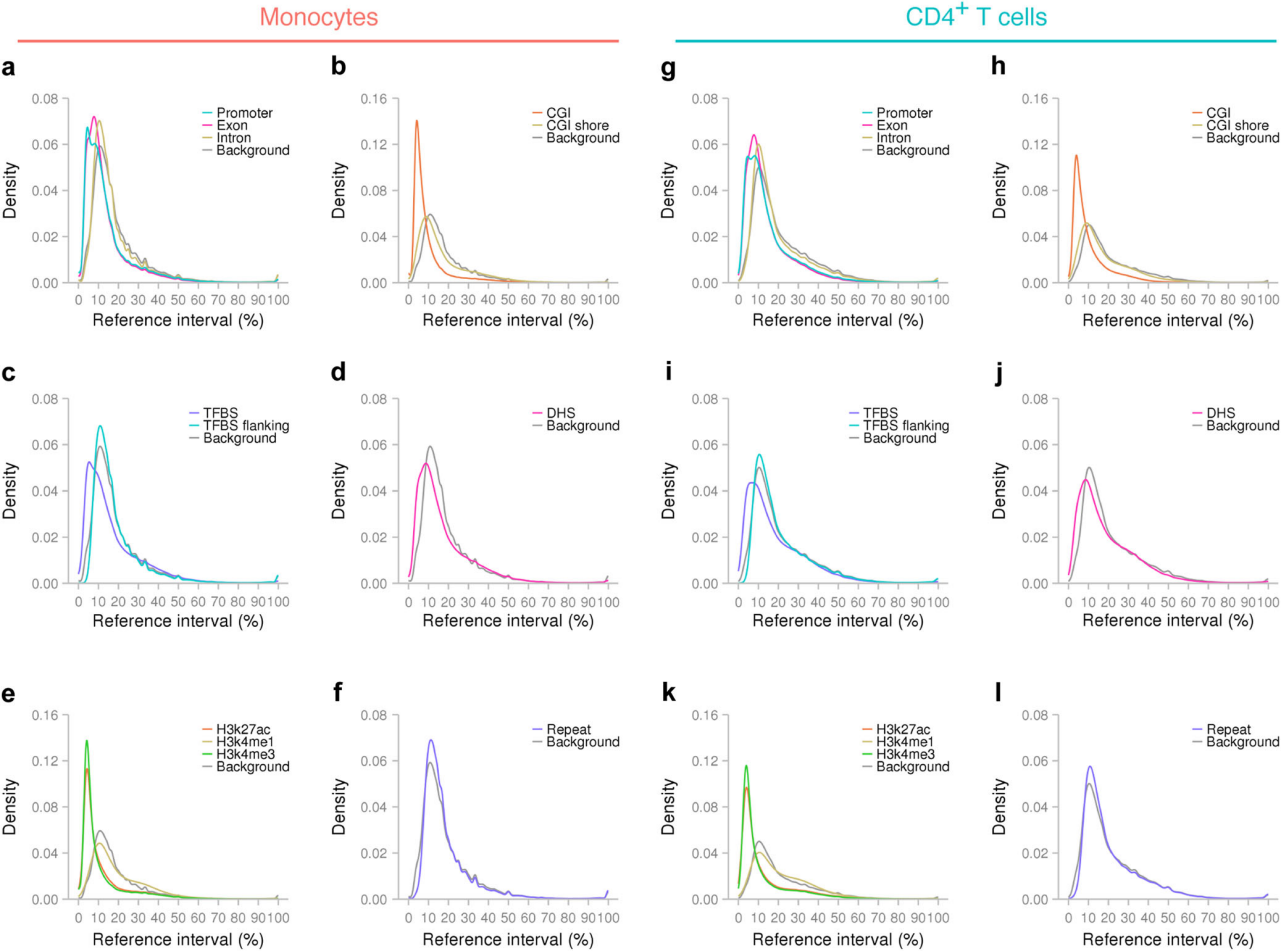
DNAm variability for regulatory elements. Distributions of reference intervals in monocytes **a**–**f** and CD4^+^ T cells **g**–**l**. Background distributions are based on the ~24 million CpG sites included in our comprehensive DNAm profiles. *CGI* CpG island, *DHS* DNase I-hypersensitive site, *H3K27ac* histone H3 acetyl Lys27, *H3K4me1* histone H3 monomethyl Lys4, *H3K4me3* histone H3 trimethyl Lys4, *TFBS* transcription factor binding site

In monocytes, regulatory elements, such as promoters (median reference interval, 10.5%), CGIs (6.4%), CGI shores (12.8%), TFBSs (12.5%), DHSs (13.0%), histone marks for active enhancers (H3K27ac; 6.9%), and histone marks for active promoters (H3K4me3; 6.2%), exhibited narrower reference intervals than the background reference interval distribution of ~24 million CpGs (14.8%) (Fig. 6a–e). Distributions of reference intervals for introns (13.9%), TFBS-flanking regions (14.5%) and repetitive regions (14.8%) were similar to the background distribution (Fig. 6a, c, e). Histone marks for (active and inactive) enhancers (H3K4me1; 15.6%) showed similar or slightly broader reference intervals (Fig. 6e). In CD4^+^ T cells, similar tendencies were observed (Fig. 6g–k).

These results revealed that DNAm levels at regulatory elements were inter-individually invariable. The generally low DNAm levels in regulatory elements (Supplementary Fig. 1) might explain the restricted DNAm variability. The notably narrow levels of reference intervals observed at active enhancers (marked by H3K27ac) and active promoters (marked by H3K4me3) suggested a strong constraint on DNAm variability in those regions. The low level of DNAm variability at the H3K27ac mark versus the relatively high variability at H3K4me1 indicated that active enhancers are specifically subject to DNAm variability constraint.

Although regional analyses of established DNAm biomarkers suggested that reference intervals for CGI shores or TFBS-flanking regions may be broad, such tendencies were not observed in the genome-wide analysis. Even for lineage-commitment TFs, such as PU.1 and PAX5, the TFBS-flanking regions did not evidently exhibit broad reference intervals (Supplementary Fig. 2).

Repetitive regions showed distributions similar to that of the background, indicating that our bioinformatics approach did not enrich for repetitive regions in a set of commonly variable CpG sites.

### Improved efficacy of the CDMV strategy

**Fig. 7:**
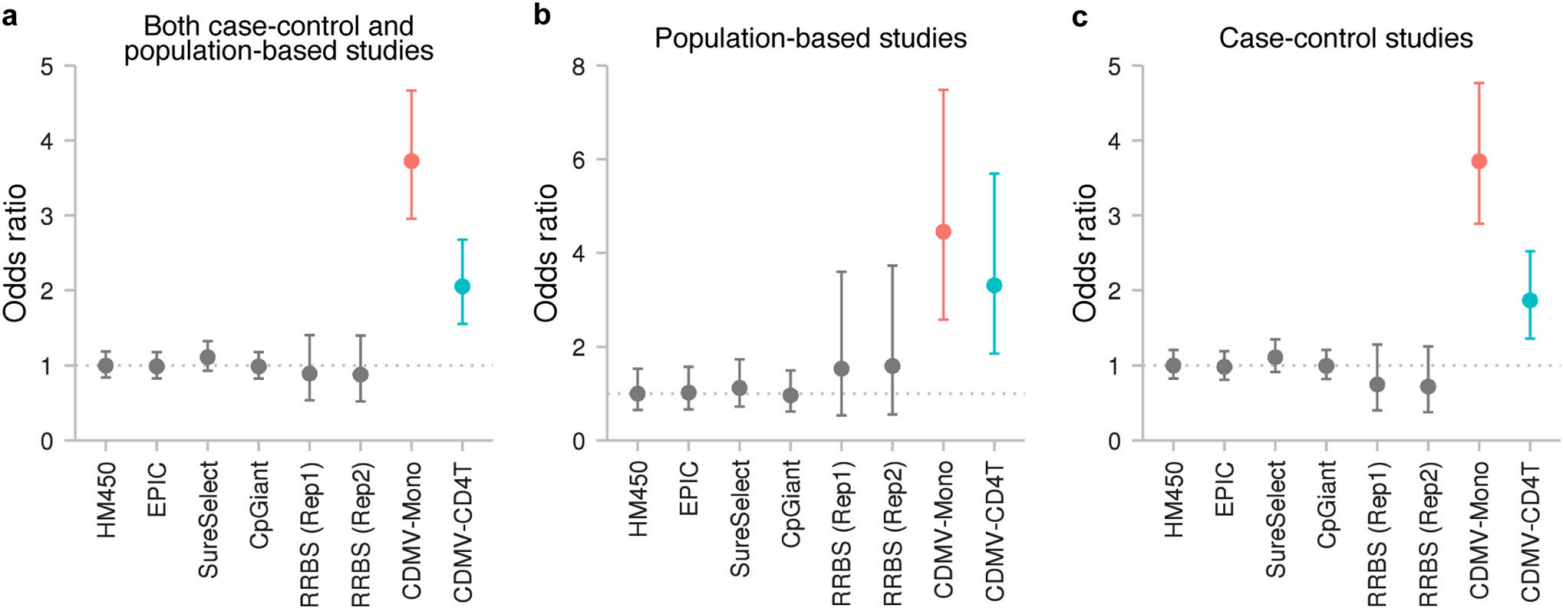
Improved efficacy of the CDMV strategy. ORs for existing designs of target CpG sites are shown as well as ORs for the strategy. CDMV-Mono and CDMV-CD4T are sets of target CpG sites determined by using the CDMV strategy from our comprehensive DNAm profiles of monocytes and CD4^+^ T cells, respectively. CDMV-Mono includes 2.0 million CpG sites and CDMV-CD4T is composed of 3.0 million CpG sites. The OR was estimated by comparing the biomarker likelihood of each set of target CpG sites with that of CpG sites probed by HM450. The 95% CIs are represented as *solid lines*. **a** ORs for population-based and case-control EWASs. The ORs were estimated based on 269 CpG sites previously identified in population-based and/or case-control EWASs. **b** ORs for population-based EWASs. The ORs were estimated based on 47 CpG sites previously identified in population-based EWASs. **c** ORs for case-control EWASs. The ORs were estimated based on 225 CpG sites previously identified in case-control EWASs. *CpGiant* SeqCap Epi CpGiant, *HM450* HumanMethylation450, *Rep* replication, *RRBS* reduced-representation bisulfite sequencing, *SureSelect* SureSelect Human Methyl-Seq

For evaluating the efficacy of the CDMV strategy, we delineated two sets of target CpG sites. The first set, CDMV-Mono, included 2.0 million CpG sites that were not located in repetitive regions and exhibited broad reference intervals (≥30%) in monocytes. Similarly, the second set, CDMV-CD4T, included 3.0 million CpG sites not located in repetitive regions and having broad reference intervals (≥30%) in CD4^+^ T cells. We excluded repetitive regions because we intended to measure the DNAm levels of those target CpG sites by microarray or methyl-capture sequencing technologies, which are unreliable for repetitive regions because of cross-hybridisation or inaccurate alignment with paralogous sequences.^19, 30^ We compared the biomarker likelihoods for CDMV-Mono and CDMV-CD4T with those for existing sets of target CpG sites. Two sets for microarrays (HM450 and EPIC), two sets for methyl-capture sequencing (SureSelect and CpGiant), and two sets profiled by RRBS experiments were considered. The number of CpG sites determined with each method is shown in Supplementary Table 12. The efficacy of epigenetic association studies was estimated by comparing the biomarker likelihood for each set of target CpG sites with the likelihood of target sites probed in the HM450 microarray, as this was the most frequently used platform in previous EWASs. Among the existing sets of target CpG sites, no set was significantly more efficient than the HM450-derived set (Fig. 7a). In contrast, as expected by the CDMV hypothesis, the two CDMV sets showed significantly improved efficacy. The OR for CDMV-Mono was 3.7 (95% confidence interval [CI]: 3.0–4.7; *P* = 1.9 × 10^−25^) and that for CDMV-CD4T was 2.1 (95% CI: 1.6–2.7; *P* = 5.2 × 10^−7^).

The two CDMV sets were derived from a population of apparently healthy subjects. We further tested whether the improved efficacy of the CDMV strategy is specific to population-based EWASs, or extendable to case-control EWASs. To evaluate the efficacy for each of case-control and population-based EWASs, we stratified the previously identified CpG sites according to study designs. Of the 269 previously identified CpG sites, 47 (17.5%) and 225 (83.6%) had been derived from population-based and case-control EWASs, respectively. Three sites had been identified in both population-based and case-control EWASs. For both study types, none of the existing sets showed a significantly improved efficacy compared to the HM450-derived set. Significantly improved efficacies of the two CDMV sets were observed for both study designs (Fig. 7b, c). The CDMV-Mono set achieved 4.5-fold (95% CI: 2.6–7.5; *P* = 1.4 × 10^−7^) and 3.7-fold (95% CI: 2.9–4.8; *P* = 1.5 × 10^−21^) improved efficacies for population-based and case-control EWASs, respectively. The efficacy of the CDMV–CD4T set for population-based EWASs (*OR* = 3.3 [95% CI: 1.9–5.7]; *P* = 3.7 × 10^−5^) was higher than that for case-control EWASs (*OR* = 1.9 [95% CI: 1.4–2.5]; *P* = 9.9 × 10^−5^).

**Fig. 8:**
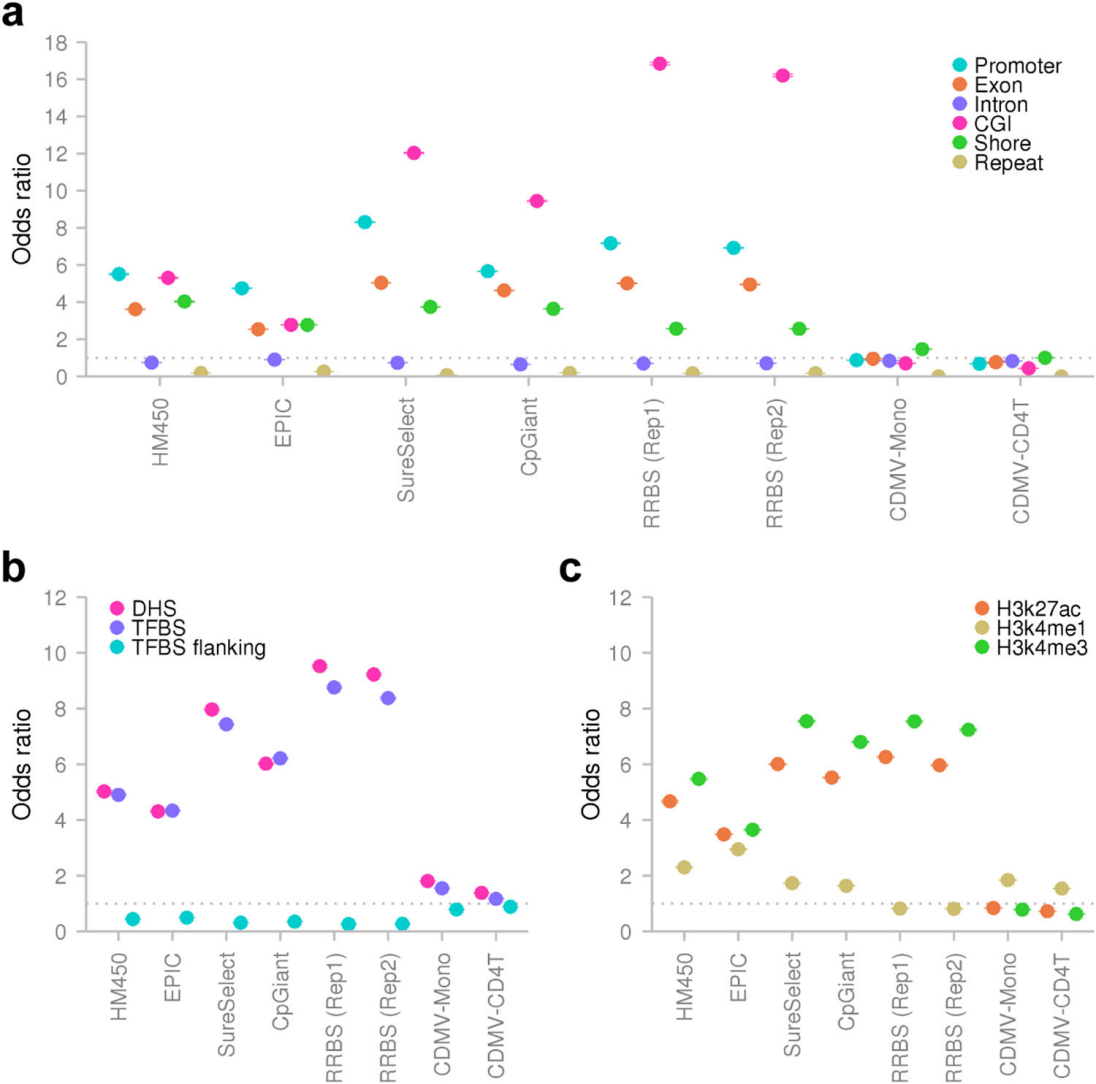
Contents of regulatory elements in previous and proposed designs of target CpG sites. Enrichment for regulatory elements in each set of target CpG sites is shown. The ORs was estimated by comparing the proportions of CpG sites that overlapped with regulatory annotations in each set. The background proportion was calculated from all CpGs in the human reference genome (hg19). *CGI* CpG island, *CpGiant* SeqCap Epi CpGiant, *DHS* DNase I-hypersensitive site, *H3K27ac* histone H3 acetyl Lys27, *H3K4me1* histone H3 monomethyl Lys4, *H3K4me3* histone H3 trimethyl Lys4, *HM450* HumanMethylation450, *Rep*, replication, *RRBS* reduced-representation bisulfite sequencing, *SureSelect* SureSelect Human Methyl-Seq, *TFBS* transcription factor binding site

**Fig. 9:**
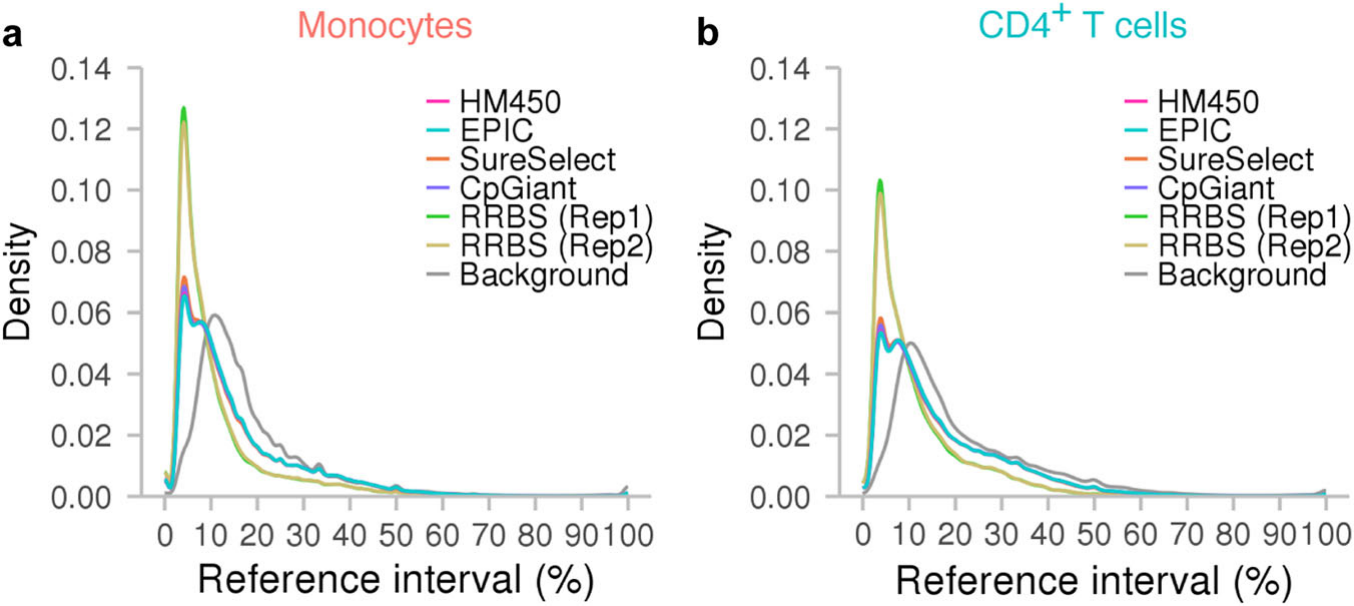
DNAm variability for target CpG sites. **a** Distributions of reference intervals in monocytes. **b** Distributions of reference intervals in CD4^+^ T cells. *CpGiant* SeqCap Epi CpGiant, *HM450* HumanMethylation450, *Rep* replication, *RRBS* reduced-representation bisulfite sequencing, *SureSelect* SureSelect Human Methyl-Seq

The existing sets of target CpG sites were enriched for regulatory elements, including promoters, CGIs, CGI shores, DHSs, TFBSs and H3K27ac and H3K4me3 marks (Fig. 8). As these regulatory elements tend to exhibit narrow reference intervals in our datasets (Fig. 6), we expected reference intervals for the CpG sites included in the existing sets to be narrow. Indeed, DNAm variability of the CpG sites targeted by the existing methods tended to be small as compared to that of the background distribution of ~24 million CpGs (Fig. 9).

These results clearly provided proof-of-concept of the improved efficacy of the CDMV strategy. Especially, CDMV-Mono achieved substantial (3.7-fold) improvement. Significantly improved efficacy was shown for case-control EWASs as well as population-based EWASs, while the efficacy for population-based EWASs was higher than that for case-control EWASs. Existing sets of target CpG sites were designed for multiple purposes; we showed that the CDMV strategy was more efficient than multipurpose designs for blood-based EWASs.

## Discussion

In this study, we tested the working hypothesis that the efficacy of epigenetic association studies may be improved by targeting inter-individually variable CpG sites. To this end, we genome widely identified commonly variable CpG sites by analysing purified monocytes and CD4^+^ T cells collected from a Japanese population of apparently healthy subjects. To estimate the efficacy of the CDMV strategy, we collected CpG sites reported by previous EWASs. Almost all previously reported CpG sites were identified from whole blood samples. Accordingly, our results implied that our catalogues of commonly variable CpG sites would improve the efficacy of future EWASs analysing whole blood samples. In addition, our results demonstrated that application of the CDMV strategy would improve the efficacy of both population-based and case-control studies. Furthermore, almost all previously reported CpG sites were identified from EWASs of Caucasians or African Americans. Therefore, the improved efficacy of our catalogues would not be restricted to EWASs of Japanese but can be extrapolated to EWASs of other ethnicities.

Our findings implicate that commonly variable CpG sites are likely to be associated with environmental exposures and/or disease susceptibilities. By taking into account the signal-to-noise ratio, CpG sites exhibiting high variability in a control group require relatively large mean differences between case and control groups to satisfy a certain *P*-value criterion as compared to CpG sites having low variability. Indeed, among 168 CpG sites associated with schizophrenia,^18^ 58 sites with broad reference intervals (≥30%) exhibited greater mean differences between case and control groups than other sites with narrow reference intervals (<30%) (Supplementary Fig. 3; *P* < 0.01, Wilcoxon rank-sum test). Meanwhile, consideration of the signal-to-noise ratio raises the possibility that CpG sites that exhibit too large a variability might not be efficient targets for future EWASs. However, the efficacy was not changed by filtering out CpG sites with reference interals of >70% from the CDMV-Mono and CDMV-CD4T catalogues (Supplementary Fig. 4). The number of CpG sites having reference intervals of >70% was only moderate (0.18 million CpGs [8.7%] in CDMV-Mono and 0.20 million CpGs [6.7%] in CDMV-CD4T), and therefore, the filtering of those CpG sites might have had little impact on the efficacy estimates.

Our catalogues of target CpG sites included 2.0 million sites in the CDMV-Mono set and 3.0 million in the CDMV-CD4T set. These numbers of CpG sites are 2 to 3 times larger than those implemented in DNAm microarrays.^19, 20, 26^ Thus, to implement our catalogues in microarrays, a further reduction of target CpG sites will be needed. However, these numbers are comparable to those targeted by methyl-capture sequencing.^29, 30^ Accordingly, implementation of the CDMV-Mono and CDMV-CD4T target CpG sites is technically possible by customizing probe sequences for methyl-capture sequencing.

We found a tight statistical link between intermediately methylated status and large inter-individual DNAm variability. The inter-individual DNAm variability was evaluated using the reference interval, which was defined in this paper as the difference between the 95th and 5th percentiles of the DNAm levels across individuals (Fig. 1b). Meanwhile, the classification of the DNAm status (i.e., hypomethylated, hypermethylated, and intermediately methylated) was determined according to the median DNAm level across our population. By definition, an intermediately methylated status does not necessarily imply a large reference interval; if all persons have the same intermediate DNAm level (i.e., 20–80%) at a CpG site, then the CpG site is classified as intermediately methylated, while the reference interval is calculated as 0%. Similarly, hypomethylated or hypermethylated status does not necessarily imply a narrow reference interval; if a CpG site is perfectly unmethylated in >50% of subjects and perfectly methylated in >5% of persons, then the CpG site is classified as unmethylated, while the reference interval is calculated as 100%. Accordingly, the link between the intermediately methylated status and large inter-individual DNAm variability can be biologically interpreted and is not just a statistical artifact.

Intermediate DNAm levels implied large cell-to-cell DNAm variability within an individual and a cell type.^56^ Accordingly, our results indicated that inter-individual DNAm variability is tightly linked to cell-to-cell DNAm variability. Further, we found that inter-individual DNAm variability at regulatory elements was strongly constrained. The constraints may act on both inter-individual and cell-to-cell DNAm variability. Consequently, genomic regions where the constraints are relaxed may show large inter-individual as well as cell-to-cell DNAm variability. Our results suggested that the molecular mechanisms behind the constraints may include histone modifications and TF binding events.

Previous epigenetics studies have revealed that processes that generate cell-to-cell DNAm variations include an imperfect DNAm transmission from mother to daughter cells,^57^ locus-specific recruitment of de novo methyltransferases (*DNMT3A* and *DNMT3B*),^58^ and demethylation by ten eleven translocation enzymes.^59^ In a recent model, locus-specific DNAm levels are regulated by multifactorial kinetics, which are affected by transmission fidelity, replication rates, de novo methyltransferase activity and demethylase activity.^60^ Our results indicate that the multifactorial kinetics would be inter-individually variable at genomic regions with balanced kinetics and thus, with intermediate DNAm levels. The kinetic balance may be shifted by in utero, childhood and adult exposures^1, 7–9^ and may be associated with intermediate phenotypes and diseases.^2, 4, 10–18^

Although studies on cell-type differences have identified outstanding switches of DNAm statuses (i.e., from hypomethylated to hypermethylated during cell differentiations),^61^ previous blood-based EWASs identified moderate shifts of DNAm levels between cases and controls.^2, 4, 7, 8, 13–18^ Even in EWASs analysing purified blood cells, inter-individual differences in DNAm levels were less dramatic than cell type-specific differences.^2, 12, 50^ The above-mentioned balanced kinetics model^60^ may explain these observations. The kinetics may be dynamically changed during cell-type differentiations involving lineage-commitment TFs and subsequent epigenetic regulation.^38^ In contrast, within a cell type, the balanced kinetics may be slightly modified in response to various environmental stimuli, which differ from person to person, while maintaining cell identity.^38, 62^

Several limitations to this study should be mentioned. Firstly, we estimated the efficacy of the CDMV strategy based on the results of previous HM450-based EWASs. This may introduce biases into the efficacy estimation. Secondly, almost all the previously reported DNAm markers were discovered from whole blood samples. Therefore, although we showed the improved efficacy of our CDMV-Mono and CDMV-CD4T catalogues for future EWASs using whole blood, we cannot state which set of commonly variable CpG sites is more effective for future EWASs using purified blood cells. Based on our data, two out of four DNAm markers previously discovered using CD4^+^ T cells exhibited broader reference intervals in CD4^+^ T cells than in monocytes, while the other two had narrower reference intervals in CD4^+^ T cells than in monocytes (Supplementary Fig. 5). In future, larger numbers of DNAm markers will be discovered using purified cells, which should allow answering the above question. Thirdly, we analysed monocytes and CD4^+^ T cells but not other blood cells, including CD8^+^ T cells, natural killer cells, B cells, and neutrophils. Fourthly, we identified commonly variable CpG sites based on a Japanese population. Since the environment can influence DNAm profiles, the geographically restricted design might cause an unintended bias in catalogues of commonly variable CpG sites. Accordingly, the efficacy of the CDMV strategy may be further improved by incorporating DNAm profiles of multiple ethnicities and of various cell types in future studies.

In conclusion, we demonstrated that the efficacy of EWASs can be improved by targeting commonly variable CpG sites. For the implementation of this efficient strategy, we provided catalogues of commonly variable CpG sites by performing WGBS-based DNAm profiling. We provided summary data for ~24 million CpGs in our web site (http://imethyl.iwate-megabank.org/downloads.html) for data sharing and future researches. Our findings and catalogues will accelerate the discovery of clinically relevant DNAm biomarkers in future EWASs.

## Methods

### Subjects

Apparently healthy subjects were enrolled from residents of the Iwate prefecture, Japan, who participated in the Tohoku Medical Megabank Community-Based Cohort Study (TMM CommCohort Study),^63^ which is being conducted by the Iwate Medical University Iwate Tohoku Medical Megabank Organisation (IMM) and the Tohoku University ToMMo. Details of the study design and recruitment method were reported previously.^63^ Of the participants in the TMM CommCohort Study, individuals visiting the Yahaba Center in the Iwate prefecture from April 2014 to June 2015 were enrolled in the present study. All participants gave written informed consent to participate in this study, which was approved by the Ethics Committee of Iwate Medical University (Approval ID: HG H25-19).

### Blood collection, FACS and DNA/RNA extraction

Peripheral blood was collected in BD Vacutainer CPT tubes containing sodium heparin (8 ml; Becton Dickinson and Company, Franklin Lakes, NJ, USA). Within 2 h after blood collection, PBMCs were collected by centrifugation (Sorvall Legend XFR; Thermo Fisher Scientific, Waltham, MA, USA) at 1,700 × *g* for 20 min at room temperature. The PBMCs were washed in 30 ml phosphate-buffered saline (PBS) containing 2 mM EDTA and then centrifuged at 250 × *g* for 10 min at room temperature to remove any contaminating platelets and plasma.

The PBMCs were incubated with CD14-FITC (catalogue #: 2228020), CD16-PE (catalogue #: 2110040), CD3-PE/Cy7 (catalogue #: 2102100) and CD4-APC (catalogue #: 2323070) antibodies (5 µl antibody/500 µl of cell suspension) (Sony Biotechnology Inc., Tokyo, Japan) for 20 min at 4°C. After washing with 5 ml of PBS, CD14^++^/CD16^−^ monocytes and CD3^+^/CD4^+^ T cells were immediately sorted using an SH800 Cell Sorter (Sony Biotechnology) from the monocyte-containing or lymphocyte-containing gate determined from light-scatter density plots (Supplementary Fig. 6). The purity of all FACS-sorted populations was analysed by flow cytometry using the SH800 Cell Sorter.

Genomic DNA and RNA were extracted from the sorted cells using the AllPrep DNA/RNA Micro Kit (Qiagen, Venlo, The Netherlands), according to the manufacturer’s instructions.

### DNAm profiling by WGBS

We carried out bisulfite conversion with the EZ DNA Methylation-Gold Kit (Zymo Research Corporation, Irvine, CA, USA) using 50 or 75 ng of genomic DNA, followed by sequencing library preparation using the TruSeq DNA Methylation Kit (Illumina Inc., San Diego, CA, USA). Fragment sizes were determined by electrophoresis on an Agilent 2200 TapeStation with D1000 ScreenTape (Agilent Technologies) and the concentration of each library was assessed by quantitative PCR with the Kapa Library Quantification Kit (Kapa Biosystems, Woburn, MA, USA) on a StepOnePlus instrument (Life Technologies, Carlsbad, CA, USA). The libraries were pooled at equimolar concentrations and loaded into flow cells with the HiSeq PE Cluster Kit v4 cBot (Illumina). The WGBS libraries were sequenced on an Illumina HiSeq 2500 instrument with the HiSeq SBS Kit v4 (paired-end 125-bp reads). To reduce the proportion of duplicated reads, we created five libraries per sample (Supplementary Table 3).

For each library, adaptor sequences were removed from raw reads using Trim Galore v0.4.0 (http://www.bioinformatics.babraham.ac.uk/projects/trim_galore/), and reads <20bp were excluded from further analyses. Then, the read sequences were mapped onto human reference genome GRCh37d5 using NovoAlign v3.02.08 (http://www.novocraft.com/) after setting the maximum alignment score acceptable for the best alignment (‘-t’ option) to 240, the strategy for reporting repeats (‘-r’ option) to ‘Random’, the homopolymer and optional dinucleotide filter score (‘-h’ option) to 120, and the bisulfite alignment mode to align reads in the forward direction using a C-to-T converted index and in the reverse complement using a G-to-A converted index (‘-b’ option). Only read pairs mapped in proper directions and within appropriate distances were retained. Duplicated amplicons were removed using SAMtools^64^ v0.1.19. The resultant bam files were merged into a single bam file for each subject.

From the merged bam files, overlaps between paired-end reads were clipped using the BamUtil package, v1.0.13 (http://genome.sph.umich.edu/wiki/BamUtil). The number of converted and unconverted cytosines in mapped reads was counted for each CpG in the human genome using NovoMethyl v3.02.08 (http://www.novocraft.com/). In this process, CpGs harbouring genetic variants in either dinucleotide were excluded. The DNAm levels were calculated for all CpGs by dividing the number of unconverted cytosines in the mapped reads by the total number of converted and unconverted cytosines in the mapped reads.

CpGs with low (<6×) and extremely high (>300×) read depths were filtered out. Only CpGs that were retained in ≥50% of the subjects were included in WGBS-based DNAm profiles for monocytes and CD4^+^ T cells.

### Gene-expression profiling by RNA-Seq

We converted 150 ng of total RNA to cDNA using Superscript II reverse transcriptase (Thermo Fisher Scientific, Waltham, MA, USA). Then, sequencing libraries were prepared using the TruSeq RNA Sample Preparation Kit v2 (Illumina). Library quality was assessed as previously described.^65^ For cluster generation with the HiSeq SR Cluster Kit v4 cBot (Illumina), six libraries were mixed in equimolar concentrations and were loaded into flow cells. Sequencing was performed in the HiSeq 2500 system (Illumina) with the HiSeq SBS Kit v4 (single-end 125-bp reads).

Read sequences were mapped onto the GRCh37 human reference genome using TopHat^66^ v2.0.13 and a guide from the Human GENCODE Gene Set (release 19).^54^ We removed reads mapped to transfer RNA and ribosomal RNA regions. Multi-mapped reads and reads with mapping quality <50 were excluded. Fragments per kb of exon per million mapped fragments values were calculated and normalised across subjects using the cuffquant and cuffnorm programs in the Cufflinks package^67^ v2.2.1.

### Genotyping by WGS

WGS was performed as previously described.^68^ Briefly, genomic DNA samples from buffy coats were fragmented using a Covaris sonicator (LE220) and subjected to library preparation with the TruSeq DNA PCR-Free HT Sample Prep Kit (Illumina). The libraries were quantified using the quantitative MiSeq method.^69^ A HiSeq 2500 system was used to generate 162-bp, paired-end reads in Rapid Run Mode with the TruSeq Rapid PE Cluster Kit and the TruSeq Rapid SBS Kit (Illumina).

Genotype data sets were constructed with the same filtering instructions used in the 1KJPN Japanese population reference panel, including single-nucleotide variant (SNV) filtering according to read coverage, software-derived biases, departures from Hardy–Weinberg equilibrium, and complexities of genomic regions around variants.^68^ For 71 samples with Japonica SNP array genotyping data,^70^ the minimum and maximum thresholds of read depth for SNV filtering were determined so as to maximise the genotype concordance between the WGS and SNP array data.^68^ For the remaining 34 samples, the minimum and maximum depth thresholds were set at 9 and 56, respectively.

### Systematic surveys for previous EWASs

We systematically searched PubMed on May 23, 2016 for EWASs that used HM450 in the discovery step and validated candidate CpG sites in independent samples, using the terms (‘epigenome wide association’) and (‘HumanMethylation450’ and ‘association’). EWASs with sample sizes smaller than 100 in the discovery step were excluded. All relevant articles were reviewed by three scientists who jointly determined for each article whether or not it satisfied our inclusion criteria.

### Statistical power for the efficacy estimation using Fisher’s exact test

We defined the biomarker likelihood for a group of CpG sites as the number of CpGs in the group that were associated with any environmental exposure and/or biomedical trait in previous EWASs divided by the number of total CpGs in the group. We estimated the efficacy of the CDMV-Mono and CDMV-CD4T catalogues by comparing the biomarker likelihoods for the two catalogues with that for the HM450 probe set using Fisher’s exact test.

The total number of autosomal CpG sites probed by the HM450 microarray was 473,814. Of these, 269 sites have been reported in previous studies. Assuming the effect size of efficacy improvement (in terms of OR) to be 2.0, 10% of the HM450 probes to be targeted, and significance level to be 0.05, statistical power was estimated as 98.6%.

### Genomic annotations for regulatory elements

Genomic coordinates for transcription start sites (TSSs), exons and introns were defined according to the Human GENCODE Gene Set (release 19).^54^ Promoter regions were determined as the regions 2kb upstream to 500bp downstream of the TSSs. Annotations for CGIs were obtained from the UCSC genome browser.^55^ CGI shores were defined as 2-kb upstream and downstream regions flanking the CGIs. Repetitive regions defined by the RepeatMasker software were retrieved from the UCSC genome browser. DHS and TFBS regions were downloaded from the UCSC ENCODE website^37, 55^ (http://genome.ucsc.edu/ENCODE/downloads.html). Annotations for three types of histone modifications (H3K27ac, H3K4me1 and H3K4me3) were retrieved from the UCSC genome browser. Annotations for histone modifications used in this study were identified based on chromatin immuno-precipitation with massively parallel sequencing of the GM12878 (a lymphoblastoid cell line produced from the blood of a female donor with northern and western European ancestry by Epstein–Barr virus transformation) and K562 (an immortalised cell line produced from a female patient with chronic myelogenous leukaemia) cell lines.

### Analysis of the potential association between DNAm level and smoking status

Smoking status (current, former or never smoker) was determined based on a self-reported questionnaire.^63^ Associations between DNAm level and smoking status were analysed with a linear-regression model with adjustments for age and sex. In this association analysis, former smokers were excluded and DNAm level differences between current and never smokers were tested. The equation for the association analysis was ${M}_{i,j}={{\beta }}_{i,0}+{{\beta }}_{i,S}{S}_{j}+{{\beta }}_{i,age}Ag{e}_{j}+{{\beta }}_{i,sex}Se{x}_{j}$, where *M*_*i,j*_ represents DNAm level for a CpG site *i* and an individual *j*, *S*_*j*_ is smoking status for an individual *j* (*S*_*j*_ = 0, never smoker; and *S*_*j*_ = 1, current smoker), *Age*_*j*_ is chronological age for an individual *j*, *Sex*_*j*_ is sex for an individual *j*, *β*_*i*,0_ is intercept for a CpG *i*, *β*_*i*,*S*_ is a coefficient for smoking status variable (expected difference between current and never smokers), *β*_*i*,*Age*_is a coefficient for age variable, and *β*_*i*,*Sex*_ is a coefficient for sex variable. DNAm level and age were regarded as continuous variables, and smoking status and sex were set as discrete variables.

### Target CpG sites in existing designs

Target CpG sites for HM450, EPIC, SureSelect and CpGiant were downloaded from the manufacturers’ websites (http://support.illumina.com/downloads.html; http://sequencing.roche.com/products/nimblegen-seqcap-target-enrichment.html and https://earray.chem.agilent.com/suredesign/, respectively). Target CpG sites for RRBS were defined according to two replicates of RRBS experiments for the GM12878 cell line. The mapping results of RRBS experiments were retrieved from the UCSC genome browser.

## Accession codes

Sequence data, DNAm profiles, gene-expression profiles, and genotypes are available upon request after approval from the Ethical Committee of Iwate Medical University, the Ethical Committee of Tohoku University, and the Materials and Information Distribution Review Committee of TMM Project. Part of the data is available as open data from the National Bioscience Database Center website (http://humandbs.biosciencedbc.jp/en) under Accession ID hum0056 and from our website (http://imethyl.iwate-megabank.org/).

## Electronic supplementary material

Supplementary Information

Supplementary Table 8

## References

[1] Rakyan VK, Down TA, Balding DJ, Beck S (2011). Epigenome-wide association studies for common human diseases. Nat. Rev. Genet..

[2] Liu Y (2013). Epigenome-wide association data implicate DNA methylation as an intermediary of genetic risk in rheumatoid arthritis. Nat. Biotechnol..

[3] Mill J, Heijmans BT (2013). From promises to practical strategies in epigenetic epidemiology. Nat. Rev. Genet..

[4] Kato N (2015). Trans-ancestry genome-wide association study identifies 12 genetic loci influencing blood pressure and implicates a role for DNA methylation. Nat. Genet..

[5] Willett WC (2002). Balancing life-style and genomics research for disease prevention. Science.

[6] Maher B (2008). Personal genomes: The case of the missing heritability. Nature.

[7] Zeilinger S (2013). Tobacco smoking leads to extensive genome-wide changes in DNA methylation. PLoS One.

[8] Tsaprouni LG (2014). Cigarette smoking reduces DNA methylation levels at multiple genomic loci but the effect is partially reversible upon cessation. Epigenetics.

[9] Argos M (2015). Gene-specific differential DNA methylation and chronic arsenic exposure in an epigenome-wide association study of adults in Bangladesh. Environ. Health Perspect..

[10] Dick KJ (2014). DNA methylation and body-mass index: a genome-wide analysis. Lancet.

[11] Demerath EW (2015). Epigenome-wide association study (EWAS) of BMI, BMI change and waist circumference in African American adults identifies multiple replicated loci. Hum. Mol. Genet..

[12] Aslibekyan S (2015). Epigenome-wide study identifies novel methylation loci associated with body mass index and waist circumference. Obesity (Silver Spring).

[13] Liang L (2015). An epigenome-wide association study of total serum immunoglobulin E concentration. Nature.

[14] Chambers JC (2015). Epigenome-wide association of DNA methylation markers in peripheral blood from Indian Asians and Europeans with incident type 2 diabetes: a nested case-control study. Lancet. Diab. Endocrinol.

[15] Florath I (2016). Type 2 diabetes and leucocyte DNA methylation: an epigenome-wide association study in over 1,500 older adults. Diabetology.

[16] Soriano-Tárraga C (2016). Epigenome-wide association study identifies TXNIP gene associated with type 2 diabetes mellitus and sustained hyperglycemia. Hum. Mol. Genet..

[17] Fasanelli F (2015). Hypomethylation of smoking-related genes is associated with future lung cancer in four prospective cohorts. Nat. Commun..

[18] Montano C (2016). Association of DNA methylation differences with schizophrenia in an epigenome-wide association study. JAMA Psychiatry.

[19] Bibikova M (2011). High density DNA methylation array with single CpG site resolution. Genomics.

[20] Moran S, Arribas C, Esteller M (2016). Validation of a DNA methylation microarray for 850,000 CpG sites of the human genome enriched in enhancer sequences. Epigenomics.

[21] Allum F (2015). Characterization of functional methylomes by next-generation capture sequencing identifies novel disease-associated variants. Nat. Commun..

[22] Shiwa Y (2016). Adjustment of cell-type composition minimizes systematic bias in blood DNA methylation profiles derived by DNA collection protocols. PLoS One.

[23] Furukawa R (2016). Intraindividual dynamics of transcriptome and genome-wide stability of DNA methylation. Sci. Rep.

[24] Andersen AM, Dogan MV, Beach SR, Philibert RA (2015). Current and future prospects for epigenetic biomarkers of substance use disorders. Genes (Basel)..

[25] Bibikova M (2009). Genome-wide DNA methylation profiling using Infinium assay. Epigenomics.

[26] Dedeurwaerder S (2011). Evaluation of the Infinium Methylation 450K technology. Epigenomics.

[27] Meissner A (2008). Genome-scale DNA methylation maps of pluripotent and differentiated cells. Nature.

[28] Walker DL (2015). DNA methylation profiling: comparison of genome-wide sequencing methods and the Infinium Human Methylation 450 Bead Chip. Epigenomics.

[29] Wang J (2011). High resolution profiling of human exon methylation by liquid hybridisation capture-based bisulfite sequencing. BMC Genomics.

[30] Li Q (2015). Post-conversion targeted capture of modified cytosines in mammalian and plant genomes. Nucleic Acids Res.

[31] Lister R (2009). Human DNA methylomes at base resolution show widespread epigenomic differences. Nature.

[32] Laurent L (2010). Dynamic changes in the human methylome during differentiation. Genome Res..

[33] Wang Q (2013). Tagmentation-based whole-genome bisulfite sequencing. Nat. Protoc..

[34] Michels KB (2013). Recommendations for the design and analysis of epigenome-wide association studies. Nat. Methods.

[35] The Cancer Genome Atlas Network (2012). Comprehensive molecular portraits of human breast tumours. Nature.

[36] Nazor KL (2012). Recurrent variations in DNA methylation in human pluripotent stem cells and their differentiated derivatives. Cell Stem Cell.

[37] ENCODE Project Consortium (2012). An integrated encyclopedia of DNA elements in the human genome. Nature.

[38] Natoli G (2010). Maintaining cell identity through global control of genomic organisation. Immunity.

[39] Irizarry RA (2009). The human colon cancer methylome shows similar hypo- and hypermethylation at conserved tissue-specific CpG island shores. Nat. Genet..

[40] Thurman RE (2012). The accessible chromatin landscape of the human genome. Nature.

[41] Grundberg E (2013). Global analysis of DNA methylation variation in adipose tissue from twins reveals links to disease-associated variants in distal regulatory elements. Am. J. Hum. Genet..

[42] van Dongen J (2014). Epigenetic variation in monozygotic twins: a genome-wide analysis of DNA methylation in buccal cells. Genes (Basel)..

[43] Gordon S, Taylor PR (2005). Monocyte and macrophage heterogeneity. Nat. Rev. Immunol..

[44] Serbina NV, Jia T, Hohl TM, Pamer EG (2008). Monocyte-mediated defense against microbial pathogens. Annu. Rev. Immunol..

[45] Chen Z (2016). Epigenomic profiling reveals an association between persistence of DNA methylation and metabolic memory in the DCCT/EDIC type 1 diabetes cohort. Proc. Natl. Acad. Sci. USA.

[46] Reynolds LM (2015). DNA Methylation of the aryl hydrocarbon receptor repressor associations with cigarette smoking and subclinical atherosclerosis. Circ. Cardiovasc. Genet.

[47] Lawrie D (2009). Local reference ranges for full blood count and CD4 lymphocyte count testing. S. Afr. Med J..

[48] Zhu J, Paul WE (2008). CD4 T cells: fates, functions, and faults. Blood.

[49] Caza T, Landas S (2015). Functional and phenotypic plasticity of CD4^+^ T cell subsets. Biomed. Res. Int.

[50] Irvin MR (2014). Epigenome-wide association study of fasting blood lipids in the genetics of lipid-lowering drugs and diet network study. Circulation.

[51] Ziller MJ, Hansen KD, Meissner A, Aryee MJ (2015). Coverage recommendations for methylation analysis by whole-genome bisulfite sequencing. Nat. Methods.

[52] Reinius LE (2012). Differential DNA methylation in purified human blood cells: implications for cell lineage and studies on disease susceptibility. PLoS One.

[53] Pfeiffer L (2015). DNA methylation of lipid-related genes affects blood lipid levels. Circ. Cardiovasc. Genet.

[54] Harrow J (2006). GENCODE: producing a reference annotation for ENCODE. Genome Biol..

[55] Karolchik D (2014). The UCSC Genome Browser database: 2014 update. Nucleic Acids Res.

[56] Elliott G (2015). Intermediate DNA methylation is a conserved signature of genome regulation. Nat. Commun..

[57] Zhao L (2014). The dynamics of DNA methylation fidelity during mouse embryonic stem cell self-renewal and differentiation. Gen. Res..

[58] Jones PA, Liang G (2009). Rethinking how DNA methylation patterns are maintained. Nat. Rev. Genet..

[59] Pastor WA, Aravind L, Rao A (2013). TETonic shift: biological roles of TET proteins in DNA demethylation and transcription. Nat. Rev. Mol. Cell Biol..

[60] Jeltsch A, Jurkowska RZ (2014). New concepts in DNA methylation. Trends Biochem. Sci..

[61] Kulis M (2015). Whole-genome fingerprint of the DNA methylome during human B cell differentiation. Nat. Genet..

[62] Vahedi G (2013). Helper T-cell identity and evolution of differential transcriptomes and epigenomes. Immunol. Rev..

[63] Kuriyama S (2016). The Tohoku Medical Megabank project: design and mission. J. Epidemiol..

[64] Li H (2009). The Sequence Alignment/Map format and SAMtools. Bioinformatics.

[65] Ohmomo H (2014). Reduction of systematic bias in transcriptome data from human peripheral blood mononuclear cells for transportation and biobanking. PLoS One.

[66] Trapnell C, Pachter L, Salzberg SL (2009). TopHat: discovering splice junctions with RNA-seq. Bioinformatics.

[67] Trapnell C (2010). Transcript assembly and quantification by RNA-seq reveals unannotated transcripts and isoform switching during cell differentiation. Nat. Biotechnol..

[68] Nagasaki M (2015). Rare variant discovery by deep whole-genome sequencing of 1,070 Japanese individuals. Nat. Commun..

[69] Katsuoka F (2014). An efficient quantitation method of next-generation sequencing libraries by using MiSeq sequencer. Anal. Biochem..

[70] Kawai Y (2015). Japonica array: improved genotype imputation by designing a population-specific SNP array with 1070 Japanese individuals. J. Hum. Genet..

