# Supplementary material for: Genome-wide identification of inter-individually variable DNA methylation sites improves the efficacy of epigenetic association studies
Source: NPJ Genom Med. 2017 Apr 13;2:11. doi: 10.1038/s41525-017-0016-5 (PMC5677974; doi:10.1038/s41525-017-0016-5)
Supplement: Supplementary file 1 — Supplementary Information [file 41525_2017_16_MOESM1_ESM.docx]

**Supplementary information for ‘Genome-wide identification of inter-individually variable DNA methylation sites improves the efficacy of epigenetic association studies’**

Supplementary figures: 1-6

Supplementary tables: 1-7 and 9-12 (Supplementary Table 8 is provided as a separate text file because of its large size)

**
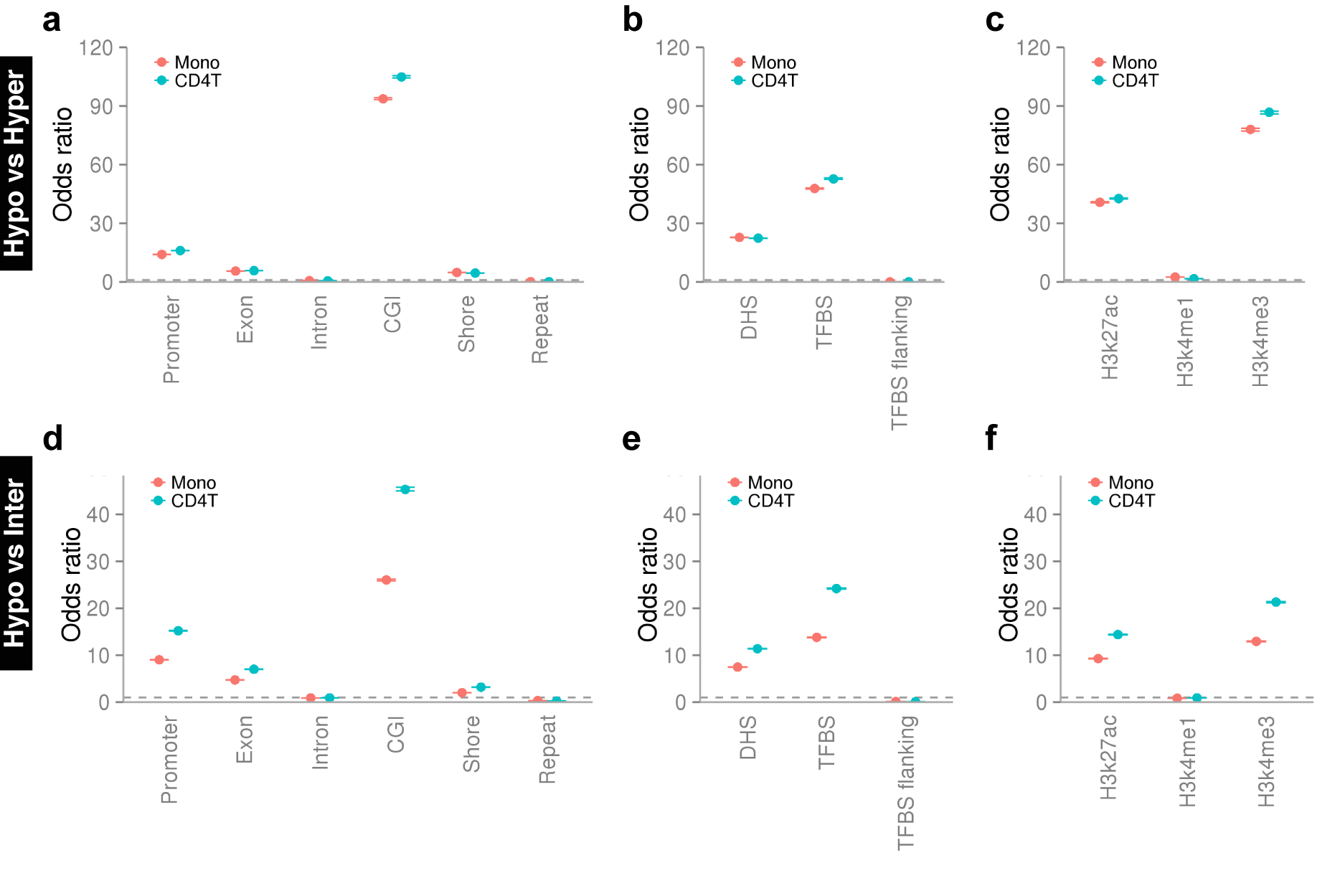
Supplementary Figure 1: Functional elements are enriched in hypomethylated regions. a–c,** Enrichment of functional elements for hypomethylated regions compared to hypermethylated regions. **d–f,** Enrichment of functional elements for hypomethylated compared to intermediately methylated regions. The results showed that functional elements, such as promoters, exons, CGIs, DHSs, TFBSs, H3K27ac modifications, and H3K4me3 modifications, are enriched for hypomethylated regions.

CGI, CpG island; DHS, DNase I-hypersensitive site; H3K27ac, histone H3 acetyl Lys27; H3K4me1, histone H3 monomethyl Lys4; H3Kme3, histone H3 trimethyl Lys4; TFBS, transcription factor binding site

**
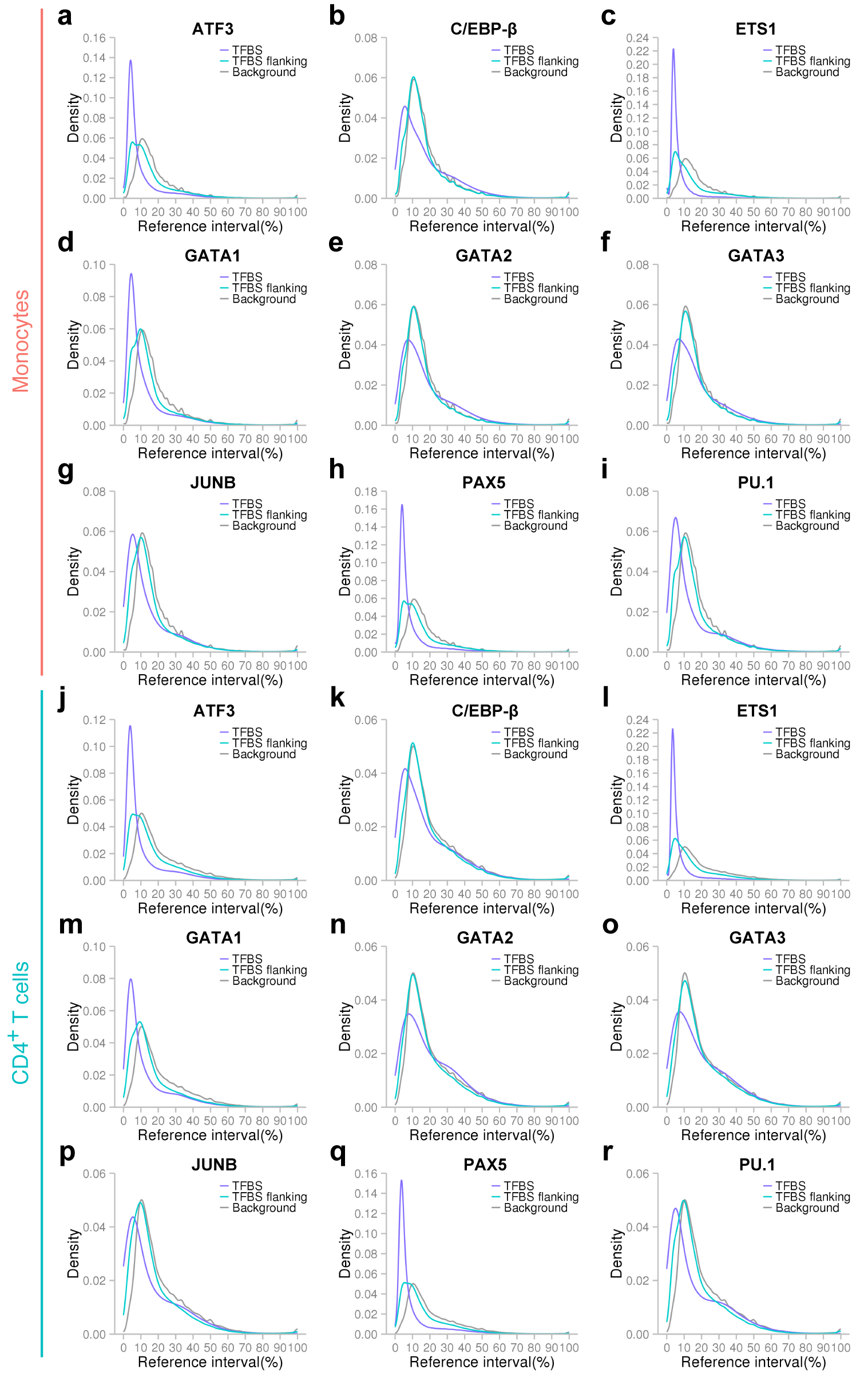
Supplementary Figure 2: Reference interval distributions for lineage-commitment transcription factor binding sites and for their flanking regions. a–i,** Distributions in monocytes. **j–r,** Distributions in CD4+ T cells.

**
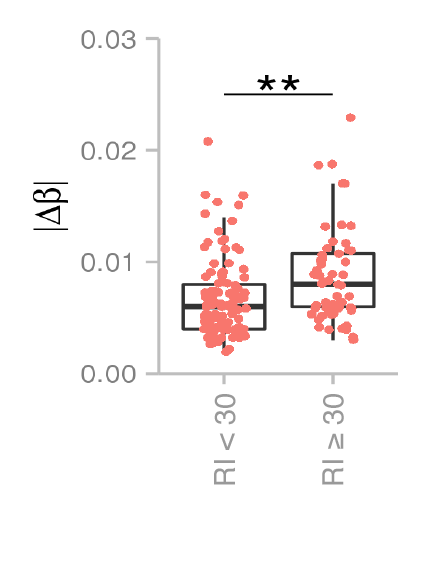
**

**Supplementary Figure 3: Difference in mean methylation levels between schizophrenia patients and control subjects (|Δβ|) for putative DNA methylation markers reported in ref. 18.** Out of 172 putative CpG sites, 58 exhibited broad reference intervals (≥30%), 110 had narrow refenrece intervals (<30%), and reference intervals for the remaining 4 sites could not estimated because the sequencing data did not pass our filtering criteria (e.g., sequencing depth or call rate). Values of |Δβ| were compared between markers exhibiting narrow monocyte-based referene intervals (<30%) and those having broad reference intervals (≥30%). RI, reference interval; **, *P* < 0.01 (Wilcoxon rank-sum test).


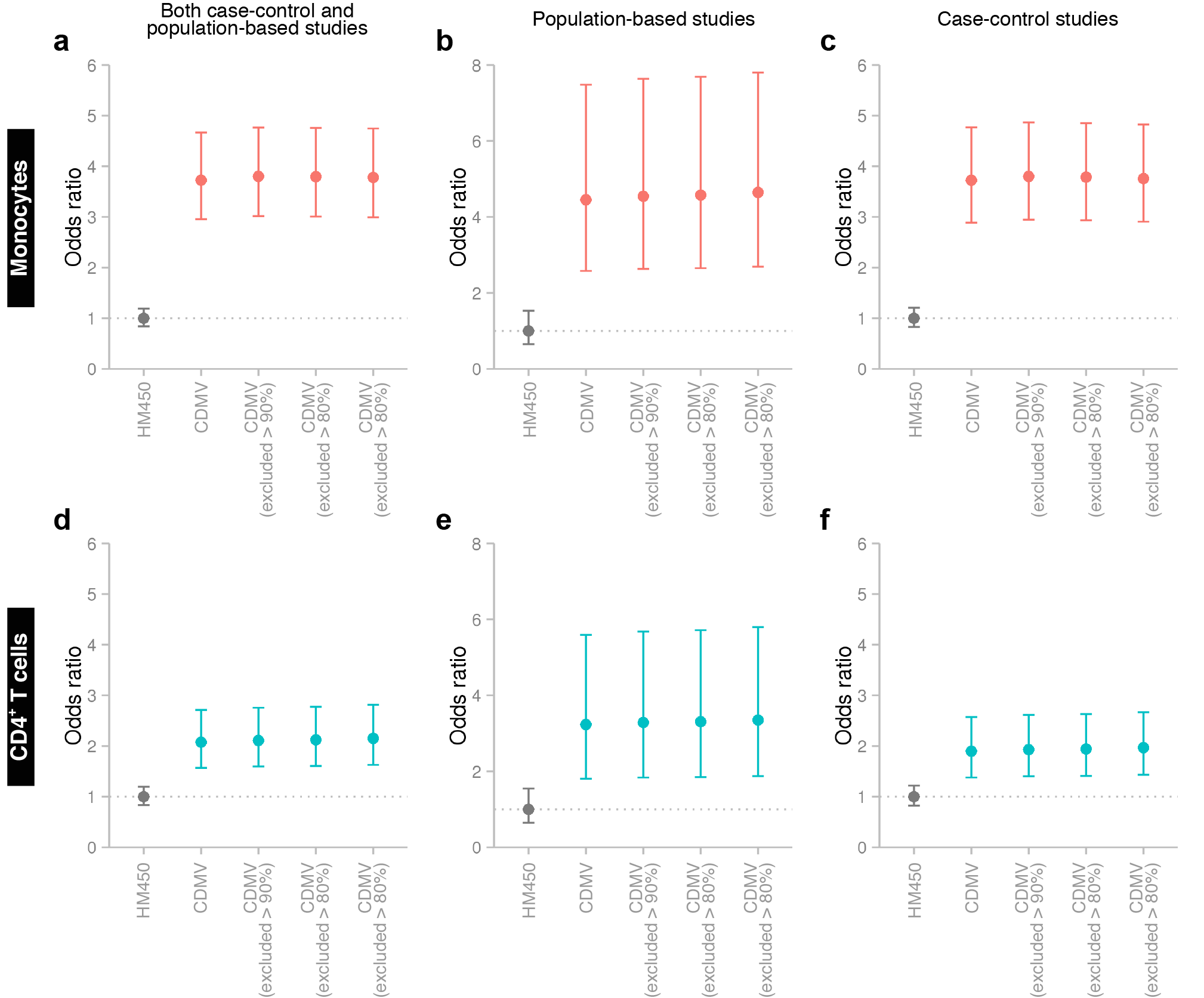


**Supplementary Figure 4: Effect of filtering out CpG sites exhibiting very large reference intervals on EWAS efficacy.** CpG sites having referene intervals of >90%, >80% and >70% were excluded from the CDMV-Mono and CDMV-CD4T catalogues. Two million CpGs were included in the CDMV-Mono set, and 0.11 (5.2%), 0.13 (6.6%), and 0.18 (8.7%) million CpGs were excluded by >90%, >80%, and >70% filtering criteria, respectively. Similarly, the CDMV-CD4T included 3.0 million CpGs, and 0.10 (3.3%), 0.14 (4.6%), and 0.20 (6.7%) million CpGs were excluded by >90%, >80% and >70% filtering criteria, respectively. The odds ratio (OR) was estimated by comparing the biomarker likelihood of each set of target CpG sites with that of CpG sites probed by HM450. The 95% confidence intervals are presented as solid lines. **a,d.** ORs for population-based and case-control EWASs. The ORs were estimated based on 269 CpG sites previously identified in population-based and/or case-control EWASs. **b,e.** ORs for population-based EWASs. The ORs were estimated based on 47 CpG sites previously identified in population-based EWASs. **c,f.** ORs for case-control EWASs. The ORs were estimated based on 225 CpG sites previously identified in case-control EWASs. CDMV, common DNAm variations.

**
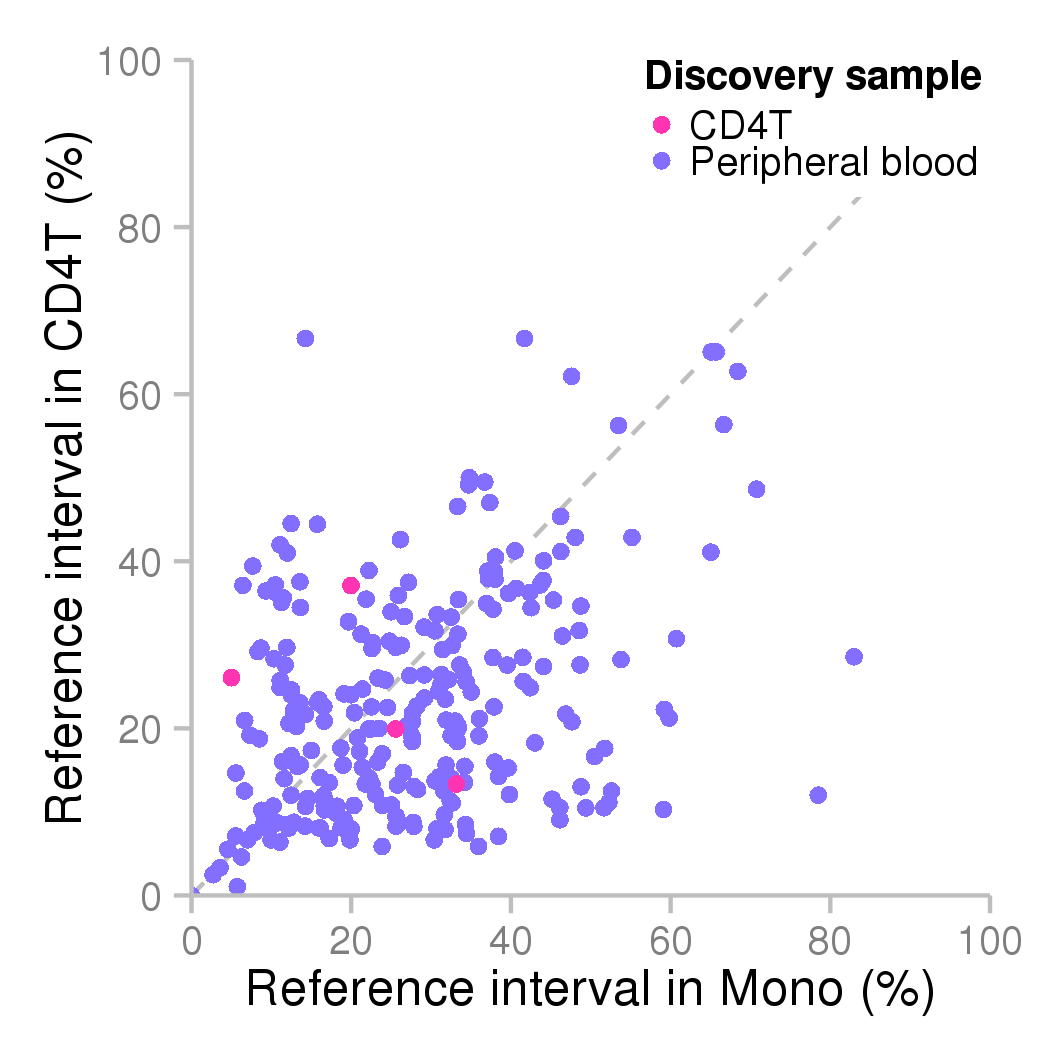
**

**Supplementary Figure 5: Monocyte- and CD4^+^ T cell-based reference intervals for putative DNA methylation markers.** Putative DNA methylation markers were obtained from previous EWASs and are listed in Supplementary Table 8. Pink dots represent putative markers discovered using CD4+ T cells in the discovery step of the previous EWASs, and blue dots indicate putative markers discovered using unsorted whole blood samples.

**
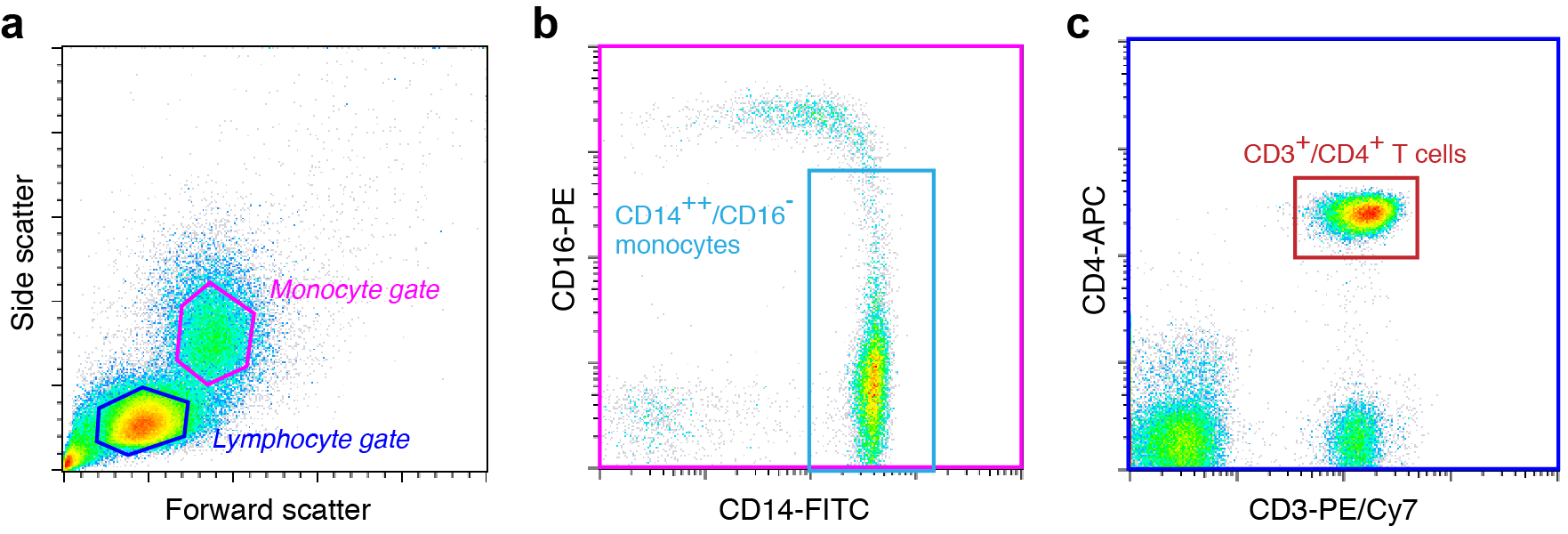
Supplementary Figure 6: Fluorescence-activated cell sorting.** **a**. Gating strategy for isolation of monocytes and lymphocytes from peripheral blood mononuclear cells (PBMCs). PBMCs were sorted according to light-scatter densities. The *x*-axis represents forward scatter density, which correlates with cell size. The *y*-axis shows side scatter density, which is proportional to cell granularity. Monocyte- and lymphocyte-containing gates are indicated as red and blue hexagons, respectively. **b.** Gating strategy for isolation of classical CD14^++^/CD16^-^ monocytes. Fluorescence densities for CD14 and CD16 are shown in the *x*- and *y*-axes, respectively. The gate for classical CD14^++^/CD16^-^ monocytes is indicated with a sky blue rectangle. **c.** Gating strategy for isolation of CD3^+^/CD4^+^ T cells. Fluorescence densities for CD3 and CD4 are shown in the *x*- and *y*-axes, respectively. The gate for CD3^+^/CD4^+^ T cells is indicated with a red rectangle.

**Supplementary Table 1: Exposures and clinical characteristics of the subjects.**

|  | Monocytes  (*N =* 102) | CD4^+^ T cells  (*N =* 102) |
| --- | --- | --- |
| Tobacco smoking |  |  |
| Current smoker, *N* (%) | 14 (13.7) | 12 (11.8) |
| Former smoker, *N* (%) | 25 (24.5) | 28 (27.5) |
| Non-smoker, *N* (%) | 63 (61.8) | 62 (60.8) |
| Alcohol use |  |  |
| Current drinker, *N* (%) | 61 (59.8) | 60 (58.8) |
| Non-drinker, *N* (%) | 41 (40.2) | 42 (41.2) |
| Alcohol consumption   in drinkers (g/week)^a^ | 184.8 ± 179.3 | 170.4 ± 193.6 |
| Sodium intake |  |  |
| Una (mEq dl^–1^)^a^ | 139.4 ± 62.9 | 141.2 ± 67.1 |
| Ucre (mg dl^–1^)^a^ | 86.1 ± 50.7 | 84.8 ± 48.6 |
| Sodium intake (g day^–1^)^a^ | 4.0 ± 0.9 | 4.0 ± 0.9 |
| Risk factors of CVD |  |  |
| Hypertension, *N* (%)^b^ | 41 (40.2) | 39 (38.2) |
| Obesity, *N* (%)^c^ | 24 (23.5) | 22 (21.6) |
| Diabetes, *N* (%)^d^ | 11 (10.8) | 10 (9.8) |
| Hypercholesterolemia, *N* (%)^e^ | 45 (44.1) | 43 (42.2) |
| CKD, *N* (%)^f^ | 13 (12.7) | 13 (12.7) |
| Medical history of CVD |  |  |
| Heart disease, *N* (%)^g,h^ | 2 (2.0) | 2 (2.0) |
| Stroke, *N* (%)^g^ | 5 (4.9) | 6 (5.9) |

^a^ Average ± standard deviation

^b^ SBP ≥ 140 mm Hg, DBP ≥ 90 mm Hg, and/or anti-hypertensive medications

^c^ BMI ≥ 25

^d^ HbA1c ≥ 6.5% and/or diabetes medications

^e^ TC ≥ 220 mg dl^–1^ and/or cholesterol-lowering medications

^f^ eGFRcre < 60 ml min^–1^ 1.73 m^–2^

^g^ Based on self-reported questionnaire

^h^ Included myocardial infarction, angina, and heart failure

BMI, body mass index; CKD, chronic kidney disease; CVD, cardiovascular disease; DBP, diastolic blood pressure; eGFRcre, glomerular filtration rate estimated from serum creatinine level; HbA1c, hemoglobin A1c; Ucre, urine creatinine concentration; UNa, urine sodium concentration; SBP, systolic blood pressure; TC, total cholesterol

**Supplementary Table 2: FACS and DNA/RNA extraction**

|  | Monocytes (*N* = 102) | CD4^+^ T cells (*N* = 102) |
| --- | --- | --- |
| Cell counts, ×10^5^ cells^a,b^ | 4.9 ± 1.4 | 6.8 ± 0.7 |
| Purity, %^a,b^ | 95.7 ± 1.5 | 96.2 ± 1.6 |
| DNA yield, ng^a,c^ | 902.6 ± 502.1 | 1,053.1 ± 499.0 |
| RNA yield, ng^a,d^ | 265.5 ± 115.7 | 204.0 ± 62.7 |

^a^ Average ± standard deviation

^b^ Analyzed by flow cytometry using an SH800 Cell Sorter (Sony Biotechnology, Tokyo, Japan)

^c^ Measured using a Qubit 2.0 Fluorometer with the Qubit dsDNA BR Kit (Life Technologies, Carlsbad, CA, USA)

^d^ Measured using a Qubit 2.0 Fluorometer and the Qubit RNA Assay Kit

FACS, fluorescence-activated cell sorting

**Supplementary Table 3: Detailed statistics for the bisulfite-treated libraries**^a^

|  |  | Monocytes (*N* = 102) | CD4^+^ T cells (*N* = 102) |
| --- | --- | --- | --- |
| Statistics for raw reads | #. Reads (denoted as ‘R1’)^b^ | 156,141,807 ± 24,547,527 | 155,842,550 ± 16,752,515 |
|  | #. Bases (denoted as ‘B1’)^b^ | 19,517,725,847 ± 3,068,440,862 | 19,480,318,794 ± 2,094,064,349 |
| Statistics after trimming adaptor sequences and removing reads shorter than 20 bp | #. Reads (denoted as ‘R2’)^b^ | 155,204,421 ± 24,486,762 | 155,132,559 ± 16,632,756 |
|  | % Reads divided by R1^b^ | 99.4 ± 1.3 | 99.5 ± 0.1 |
| Statistics for mapped reads | #. Reads (denoted as ‘R3’)^b^ | 141,489,208 ± 22,540,537 | 140,771,766 ± 15,212,394 |
|  | % Reads divided by R2^b^ | 91.1 ± 2.0 | 90.7 ± 1.1 |
| Statistics after removing duplicated PCR amplicons | #. Reads^b^ | 124,886,574 ± 26,613,434 | 133,586,866 ± 14,044,851 |
|  | % Reads divided by R3^b^ | 88.2 ± 13.0 | 94.9 ± 0.9 |
|  | #. Bases^b^ | 13,740,571,211 ± 3,092,922,224 | 14,970,990,757 ± 1,562,162,615 |
|  | % Bases divided by B1^b^ | 70.4 ± 15.8 | 76.9 ± 8.0 |
|  | Average of insert lengths, bp^b^ | 165 ± 10 | 172 ± 7 |

^a^ Five libraries were prepared and sequenced for each sample

^b^ Average ± standard deviation

**Supplementary Table 4: Detailed statistics for WGBS quality-control filtering**

|  |  | Monocytes (*N* = 102) | CD4^+^ T cells (*N* = 102) |
| --- | --- | --- | --- |
| Statistics for raw reads | # Reads (denoted as ‘R1’)^a^ | 780,709,034 ± 45,934,514 | 779,212,752 ± 40,833,955 |
|  | # Bases (denoted as ‘B1’)^a^ | 97,588,629,235 ± 5,741,814,291 | 97,401,593,968 ± 5,104,244,342 |
|  | Depth^a^ | 31.1 ± 1.8 | 31.0 ± 1.6 |
| Statistics after trimming adaptor sequences, mapping, and removing duplicated PCR amplicons | # Reads (denoted as ‘R2’)^a^ | 624,432,868 ± 38,766,158 | 667,934,331 ± 33,002,407 |
|  | % Reads divided by R1^a^ | 80.0 ± 5.0 | 85.7 ± 4.2 |
|  | # Bases (denoted as ‘B2’)^a^ | 68,702,856,057 ± 4,522,417,669 | 74,854,953,783 ± 3,497,433,720 |
|  | % Bases divided by B1^a^ | 70.4 ± 4.6 | 76.9 ± 3.6 |
| Statistics after clipping overlaps between paired reads | # Reads^a^ | 624,432,868 ± 38,766,158 | 667,934,331 ± 33,002,407 |
|  | % Reads divided by R2^a^ | 100.0 ± 0.0 | 100.0 ± 0.0 |
|  | # Bases^a^ | 47,245,324,384 ± 3,327,997,912 | 52,472,000,722 ± 2,376,329,508 |
|  | % Bases divided by B2^a^ | 68.8 ± 0.9 | 70.1 ± 0.7 |
|  | Depth^a^ | 15.1 ± 1.1 | 16.7 ± 0.8 |

^a^ Average ± standard deviation

**Supplementary Table 5: RNA-seq data statistics**

|  |  | Monocytes (*N* = 102) | CD4^+^ T cells (*N* = 102) |
| --- | --- | --- | --- |
| Sequencing statistics | # Raw reads (denoted as ‘R1’)^a^ | 33,917,157 ± 3,153,528 | 35,175,996 ± 1,275,575 |
|  | # Mapped reads (denoted as ‘R2’)^a^ | 29,702,870 ± 2,740,121 | 30,729,363 ± 1,875,638 |
|  | % Mapped reads divided by R1^a^ | 87.6 ± 2.9 | 87.3 ± 3.9 |
|  | # Reads after removing rRNA/tRNA-derived and low mapping-quality reads^a^ | 27,390,039 ± 2,494,286 | 27,506,624 ± 1,669,459 |
|  | % Reads after removing rRNA/tRNA-derived and low mapping quality reads divided by R2^a^ | 92.2 ± 0.3 | 89.5 ± 0.6 |
| Gene expression statistics | # Genes annotated by GENCODE release 19 | 57,815 | 57,815 |
|  | # Genes expressed with FPKM ≥ 0.1^a^ | 11,746 ± 68 | 13,063  ± 89 |
|  | % Genes expressed with FPKM ≥ 0.1^a^ | 20.3  ± 0.1 | 22.6  ± 0.2 |
|  | # Genes expressed with FPKM ≥ 0.1 in ≥ 50% of subjects | 16,789 | 18,894 |
|  | % Genes expressed with FPKM ≥ 0.1 in ≥ 50% of subjects | 29.0 | 32.7 |

^a^ Average ± standard deviation

FPKM, fragments per kilobase of exon per million mapped fragments; RNA-Seq, RNA sequencing

**Supplementary Table 6: WGS data statistics**

|  |  | WGS subjects |
| --- | --- | --- |
| Sequencing statistics | Total samples | 105 |
|  | Total raw bases | 9.8 trillion bases |
| Statistics for high-confidence SNVs | Total SNVs | 9,619,480 |
|  | Number of known variants | 7,504,553 |
|  | Number of novel variants^a^ | 2,114,927 |
|  | Novelty rate^a^ | 21.99% |
|  | Average number per sample | 2,795,534 |
|  | Average individual heterozygosity | 1,569,772 |

^a^ Comparison to dbSNP build 138

SNV, single-nucleotide variants; WGS, whole-genome sequencing

**Supplementary Table 7: Previous EWASs used to estimate efficacy for future epigenetic association studies**

| Trait | Discovery | | | |  | Replication | | | |  |
| --- | --- | --- | --- | --- | --- | --- | --- | --- | --- | --- |
|  | Race/Ethnicity | Target cell | Study design  (#. Individuals) | #. CpG |  | Race/Ethnicity | Target cell | Study design  (#. Individuals) | #. CpG | PubMed  ID |
| Smoking exposure | Caucasians | Whole blood | Case-control  (262 – 749) | 972 |  | Caucasians | Whole blood | Case-control  (236 – 232) | 32 | 23691101 |
| Smoking exposure | Caucasians | Whole blood | Case-control  (22 – 179) | 53 |  | Caucasians | Whole blood | Case-control  (41 – 211) | 29 | 25424692 |
| BMI | African Americans | Whole blood | Population-based  (2097) | 76 |  | Caucasians | Whole blood | Population-based  (2377) | 37 | 25935004 |
| BMI | Caucasians | CD4^+^ T cell | Population-based  (991) | 8 |  | African Americans | Whole blood | Population-based  (2097) | 3 | 26110892 |
| BMI | Caucasians | Whole blood | Population-based  (239) | 3 |  | Caucasians | Whole blood | Population-based  (339) | 3 | 24630777 |
| WC | African Americans | Whole blood | Population-based  (2097) | 164 |  | Caucasians | Whole blood | Population-based  (2377) | 8 | 25935004 |
| WC | Caucasians | CD4^+^ T cell | Population-based  (991) | 5 |  | African Americans | Whole blood | Population-based  (2097) | 4 | 26110892 |
| Type 2 diabetes | Indian Asians | Whole blood | Case-control  (1074 – 1590) | 7 |  | Caucasians | Whole blood | Case-control  (377 – 764) | 5 | 26095709 |
| Type 2 diabetes | Caucasians | Whole blood | Case-control  (151 – 204) | 4 |  | Caucasians | Whole blood | Case-control  (122 – 690) | 1 | 26643952 |
| Type 2 diabetes | Caucasians | Whole blood | Case-control  (69 – 835) | 39 |  | Caucasians | Whole blood | Case-control  (39 – 440) | 1 | 26433941 |
| TG | Caucasians | Whole blood | Population-based  (1776) | 10 |  | Caucasians | Whole blood | Population-based  (499) | 7 | 25583993 |
| TG | Caucasians | CD4^+^ T cell | Population-based  (991) | 4 |  | Caucasians | Whole blood | Population-based  (2846) | 1 | 24920721 |
| HDL-C | Caucasians | Whole blood | Population-based  (1776) | 1 |  | Caucasians | Whole blood | Population-based  (499) | 1 | 25583993 |
| LDL-C | Caucasians | Whole blood | Population-based  (1776) | 1 |  | Caucasians | Whole blood | Population-based  (499) | 1 | 25583993 |
| VLDL-C | Caucasians | CD4^+^ T cell | Population-based  (991) | 4 |  | Caucasians | Whole blood | Population-based  (2846) | 1 | 24920721 |
| Schizophrenia | Multi-ethnicities^a^ | Whole blood | Case-control  (689 – 645) | 923 |  | Multi-ethnicities^a^ | Whole blood | Case-control  (247 – 250) | 172 | 27074206 |

**^a^** Almost subjects were Caucasians or African Americans

BMI, body mass index; WC, waist circumference; TG, triglyceride; HDL-C; high-density lipoprotein cholesterol; LDL-C, low-density lipoprotein cholesterol; VLDL-C , very-low-density lipoprotein cholesterol

**Supplementary Table 9: Associations between DNAm and gene-expression levels at established biomarker loci**

| CpG | Gene(s) | Chr | Position | Cell type | Association with exposure | |  | Association with expression | |
| --- | --- | --- | --- | --- | --- | --- | --- | --- | --- |
|  |  |  |  |  | *P* | Δβ, % (95% CI)^a^ |  | *P* | Δlog_10_[FPKM+0.1], × 10^-2^ (95% CI)^b^ |
| cg05575921 | *AHRR* | 5 | 373,378 | Monocytes | **4.8 × 10^-3^** | **-16.4 (-27.7 – -5.1)** |  | **1.1 × 10^-5^** | **-0.8 (-1.2 – -0.5)** |
|  |  |  |  | CD4^+^ T | 0.78 | -0.6 (-5.0 – 3.8) |  | 0.13 | -0.4 (-1.0 – 0.1) |
| cg03636183 | *F2RL3* | 19 | 17,000,585 | Monocytes | **3.7 × 10^-3^** | **-8.3 (-13.9 – -2.8)** |  | 0.30 | -0.1 (-0.4 – 0.1) |
|  |  |  |  | CD4^+^ T | 0.17 | -2.0 (-4.8 – 0.9) |  | 0.20 | 0.5 (-0.3 – 1.3) |

Results listed in bold are nominally significant.

^a^ Difference in DNA methylation levels (β) between current smokers and never smokers

^b^ Difference in log10[FPKM + 0.1] level per 1% change of DNA methylation level

CI, confidence interval; FPKM, fragments per kilobase of exon per million mapped fragments

**Supplementary Table 10: Associations of genetic variants neighboring cg05575921 with DNAm level and smoking status**

| ID | Chr | Position | REF | ALT | MAF | Association with DNAm level | |  | Association with tobacco exposure | |
| --- | --- | --- | --- | --- | --- | --- | --- | --- | --- | --- |
|  |  |  |  |  |  | *P* | Δβ, % (95% CI) |  | *P* | Odds ratio, % (95% CI) |
| rs908114 | 5 | 371958 | C | T | 0.37 | 0.18 | -3.7 (-9.2 – 1.8) |  | 0.18 | 0.5 (0.2 – 1.3) |
| rs373918364 | 5 | 374088 | A | G | 0.02 | 0.80 | 2.5 (-16.9 – 21.9) |  | 0.99 | 9.6 × 10^-8^ (0.0 – Inf) |
| rs1877843 | 5 | 374510 | C | T | 0.36 | 0.29 | -3.0 (-8.7 – 2.6) |  | 0.10 | 0.4 (0.1 – 1.2) |
| rs1877842 | 5 | 374532 | G | A | 0.36 | 0.29 | -3.0 (-8.7 – 2.6) |  | 0.12 | 0.4 (0.2 – 1.2) |
| rs1877840 | 5 | 374748 | A | G | 0.37 | 0.18 | -3.8 (-9.5 – 1.8) |  | 0.19 | 0.5 (0.2 – 1.4) |
| rs4957030 | 5 | 374951 | G | A | 0.37 | 0.32 | -2.8 (-8.3 – 2.8) |  | 0.18 | 0.5 (0.2 – 1.3) |
| rs4957031 | 5 | 375096 | C | T | 0.37 | 0.17 | -3.8 (-9.3 – 1.7) |  | 0.20 | 0.5 (0.2 – 1.4) |
| rs2671912 | 5 | 375107 | T | C | 0.38 | 0.25 | -3.2 (-8.7 – 2.3) |  | 0.13 | 0.5 (0.2 – 1.2) |

ALT, alternative allele; Chr, chromosome; CI, confidence interval; DNAm, DNA methylation; MAF, minor allele frequency; REF, reference allele

**Supplementary Table 11: Associations of genetic variants neighboring cg03636183 with DNAm level and smoking status**

| ID | Chr | Position | REF | ALT | MAF | Association with DNAm level | |  | Association with tobacco exposure | |
| --- | --- | --- | --- | --- | --- | --- | --- | --- | --- | --- |
|  |  |  |  |  |  | *P* | Δβ, % (95% CI) |  | *P* | Odds ratio, %  (95% CI) |
| rs56298289 | 19 | 16998719 | G | A | 0.04 | 0.79 | -1.0 (-8.9 – 6.8) |  | 1.00 | 1.0 (0.1 – 9.7) |
| rs773905 | 19 | 16999897 | T | C | 0.28 | 0.10 | 2.4 (-0.5 – 5.3) |  | 0.64 | 1.2 (0.5 – 2.9) |
| rs773904 | 19 | 17000131 | G | A | 0.12 | 0.68 | -0.9 (-5.3 – 3.5) |  | 0.76 | 1.2 (0.3 – 4.5) |
| rs773903 | 19 | 17000231 | C | T | 0.11 | 0.68 | -1.0 (-5.6 – 3.7) |  | 0.48 | 1.6 (0.4 – 6.1) |
| rs773902 | 19 | 17000632 | G | A | 0.21 | 0.57 | 1.0 (-2.4 – 4.3) |  | 0.70 | 1.2 (0.5 – 3.1) |
| rs190167952 | 19 | 17000778 | C | T | 0.05 | 0.55 | -2.0 (-8.4 – 4.5) |  | 0.10 | 3.9 (0.8 – 19.8) |
| rs2227349 | 19 | 17001551 | G | A | 0.07 | 0.02 | 6.3 (0.9 – 11.7) |  | 0.77 | 1.3 (0.2 – 7.0) |

ALT, alternative allele; Chr, chromosome; CI, confidence interval; DNAm, DNA methylation; MAF, minor allele frequency; REF, reference allele

**Supplementary Table 12: Number of CpG sites in each design of target CpG sites**

| Design of target CpG sites | # CpGs |
| --- | --- |
| HumanMethylation450K | 461,020 |
| MethylationEPIC | 1,792,180 |
| SureSelect Human Methyl-Seq | 3,011,192 |
| NimbleGen SeqCap Epi CpGiant | 2,727,124 |
| Reduced-representation bisulfite enzyme *Msp*I (Replication 1) | 1,144,259 |
| Reduced-representation bisulfite enzyme *Msp*I (Replication 2) | 1,110,609 |
| CDMV-Mono | 2,018,583 |
| CDMV-CD4T | 2,979,947 |
